# Retina Organoid Transplants Develop Photoreceptors and Improve Visual Function in RCS Rats With RPE Dysfunction

**DOI:** 10.1167/iovs.61.11.34

**Published:** 2020-09-18

**Authors:** Bin Lin, Bryce T. McLelland, Robert B. Aramant, Biju B. Thomas, Gabriel Nistor, Hans S. Keirstead, Magdalene J. Seiler

**Affiliations:** 1 Physical Medicine & Rehabilitation, Sue & Bill Gross Stem Cell Research Center, University of California at Irvine, School of Medicine, Irvine, California, United States; 2 USC Roski Eye Institute, Department of Ophthalmology, University of Southern California, Los Angeles, California, United States; 3 AIVITA Biomedical Inc., Irvine, California, United States; 4 Ophthalmology, University of California at Irvine, School of Medicine, Irvine, California, United States; 5 Anatomy & Neurobiology, University of California at Irvine School of Medicine, Irvine, California, United States

**Keywords:** RPE dysfunction, retinal degeneration, human embryonic stem cell, electroretinogram, optokinetic testing, superior colliculus electrophysiology, optical coherence tomography

## Abstract

**Purpose:**

To study if human embryonic stem cell–derived photoreceptors could survive and function without the support of retinal pigment epithelium (RPE) after transplantation into Royal College of Surgeons rats, a rat model of retinal degeneration caused by RPE dysfunction.

**Methods:**

CSC14 human embryonic stem cells were differentiated into primordial eye structures called retinal organoids. Retinal organoids were analyzed by quantitative PCR and immunofluorescence and compared with human fetal retina. Retinal organoid sheets (30–70 day of differentiation) were transplanted into immunodeficient RCS rats, aged 44 to 56 days. The development of transplant organoids in vivo in relation to the host was examined by optical coherence tomography. Visual function was assessed by optokinetic testing, electroretinogram, and superior colliculus electrophysiologic recording. Cryostat sections were analyzed for various retinal, synaptic, and donor markers.

**Results:**

Retinal organoids showed similar gene expression to human fetal retina transplanted rats demonstrated significant improvement in visual function compared with RCS nonsurgery and sham surgery controls by ERGs at 2 months after surgery (but not later), optokinetic testing (up to 6 months after surgery) and electrophysiologic superior colliculus recordings (6–8 months after surgery). The transplanted organoids survived more than 7 months; developed photoreceptors with inner and outer segments, and other retinal cells; and were well-integrated within the host.

**Conclusions:**

This study, to our knowledge, is the first to show that transplanted photoreceptors survive and function even with host's dysfunctional RPE. Our findings suggest that transplantation of organoid sheets from stem cells may be a promising approach/therapeutic for blinding diseases.

Retinal degeneration (RD) diseases such as retinitis pigmentosa, Stargardt disease, and age-related macular degeneration (AMD) are major causes of blindness owing to photoreceptor degeneration. The RPE has several functions, including light absorption, epithelial transport, spatial ion buffering, visual cycle, phagocytosis, secretion, and immune modulation.^1^ Photoreceptor outer segments tips are shed daily and phagocytosed by RPE cells, which recycle metabolites to photoreceptors. If RPE phagocytosis fails, as in the Royal College of Surgeons (RCS) rat,^2^^,^^3^ outer segment debris accumulates and results in photoreceptor death. At early RD disease stages, damaged photoreceptors can be rescued by treatments, such as gene therapy,^4^^,^^5^ and trophic factors.^6^^,^^7^ However, photoreceptors are irreversibly damaged with further disease advancement and cannot be rescued. Retinal ganglion cells can survive after severe photoreceptor loss,^8^ which suggests that replacement of photoreceptors may restore vision.

Fetal retinal sheet transplants replace degenerating retinal cells and improve visual function, which demonstrates that the mature retina can incorporate new photoreceptors into preexisting circuitry.^9^^–^^13^ However, fetal retina tissues are difficult to scale to a clinically relevant level and may not be available for clinical trials owing to ethical concerns.

Stem cells have been considered a promising source for cell therapy.^14^ Pluripotent stem cells, such as human embryonic stem cells (hESCs) and induced pluripotent stem cells, can be differentiated into three-dimensional retinal organoids with similar properties as fetal retina.^15^^–^^19^ Although stem cell–derived transplants improved visual function, transplants only formed rosettes (spherical arrangement of retinal layers, with photoreceptors in the center and separated from RPE).^20^^–^^22^ Furthermore, it has been shown that both photoreceptors and RPE were necessary for optimal transplant photoreceptor organization.^10^^,^^23^^,^^24^ Many retinal diseases include both RPE dysfunction and photoreceptor degeneration. To restore vision in those patients, both RPE and photoreceptors should be replaced. However, this goal is difficult to achieve with stem cell-derived tissues because RPE and retina organoids have different media requirements. Thus, one interesting question will be whether RPE is needed for the photoreceptors in the transplant to function normally. If the transplanted photoreceptors can survive and function in the presence of dysfunctional host RPE, they may restore vision not only in patients with photoreceptor degeneration, but also in patients with RPE dysfunction.

The RCS rat is a model of photoreceptor degeneration owing to RPE dysfunction^2^, caused by a deletion in the Mer tyrosine kinase (*MerTK*) receptor that abolishes RPE cell phagocytosis.^3^^,^^25^ Most photoreceptors are lost at 2 to 3 months of age, resulting in severely impaired retinal function.^26^^–^^28^ RPE cells,^29^^–^^31^ and other cell types,^32^^–^^34^ can rescue photoreceptors when transplanted at early stages (P21–28), but not at later stages of degeneration (P42).^25^ Therefore, we investigated whether transplanted photoreceptors can survive and function in a rat model of RPE dysfunction by transplanting retinal organoid sheets at a later stage (P44–56). Furthermore, nude immunodeficient RCS rats were used, which do not reject xenografts owing to lack of T cells.^35^

## Methods

### Cell Culture

CSC-14 hESCs (AIVITA Biomedical Inc., Irvine, CA; National Institutes of Health [NIH] registered line 0284) were grown and expanded using a chemically defined and xeno-free custom formulated media (Irvine Scientific, Irvine, CA) supplemented with low levels of bovine fibroblast growth factor and activin-A. Cells were grown on thin Matrigel (Corning, Corning, NY) and passaged every 3-4 days at 1:6 to 1:10 splits using Collagenase IV digestion. Retinal organoids were generated using a protocol previously described.^19^^,^^22^

### Quantitative Polymerase Chain Reaction (qPCR)

A custom qPCR array (Qiagen, Germantown, MD; Cat# CAPH13339; 84 retinal genes and 5 housekeeping genes) was used to analyze the expression pattern in retinal organoids and control human fetal retinal tissues. Fetal control tissues (*n* = 7) ranged from day 105 to 145 and were obtained from Human Stem Cell Research Oversight Committee–approved suppliers. Differentiated retinal organoids were analyzed at day 37 to 70 (*n* = 8). Most samples analyzed were postprocessed pieces remaining from organoids used to dissect retinal sheets for transplantation. The genes analyzed are listed in Table 1. RNA was isolated using Trizol reagent (Qiagen), DNase I digested (Thermo Fisher, Waltham, MA), and phenol:chloroform extraction (Thermo Fisher). cDNA was generated using RT^2^ cDNA synthesis kit (Qiagen). Amplification was performed using RT^2^ Sybr Green with ROX qPCR master mix (Qiagen), with the following cycling conditions: 95°C (10 minutes); followed by 40 cycles of 95°C (1 minute), and 60°C (30 seconds). Cycle threshold (Ct) values were determined using Viia7 RUO software (ThermoFisher). Delta Ct values were calculated using RPL7 as the housekeeping gene. The mean Delta Ct value per gene was determined and scatterplots of mean delta Ct values for human fetal retina vs retinal organoids was plotted. Both the “hclust” R-program algorithm to analyze qPCR data and code used to create the gene array scatterplots were downloaded from The R Foundation (https://www.r-project.org/).

**Table 1.** List of Genes in Gene Array

Gene ID	Accession Number	Official Full Name
*BEST1*	NM_004183	Bestrophin 1
*CRX*	NM_000554	Cone-rod homeobox
*GLUL*	NM_002065	Glutamate-ammonia ligase
*LHX2*	NM_004789	LIM homeobox 2
*MITF*	NM_000248	Microphthalmia-associated transcription factor
*NRL*	NM_006177	Neural retina leucine zipper
*POU5F1*	NM_002701	POU class 5 homeobox 1
*OTX2*	NM_021728	Orthodenticle homeobox 2
*OPN1LW*	NM_020061	Opsin 1 (cone pigments), long-wave-sensitive
*PAX6*	NM_000280	Paired box 6
*PRKCB*	NM_002738	Protein kinase C, beta
*RAX*	NM_013435	Retina and anterior neural fold homeobox
*RCVRN*	NM_002903	Recoverin
*RHO*	NM_000539	Rhodopsin
*RLBP1*	NM_000326	Retinaldehyde binding protein 1
*RPE65*	NM_000329	RPE-specific protein 65kDa
*SIX3*	NM_005413	SIX homeobox 3
*SIX6*	NM_007374	SIX homeobox 6
*SOX2*	NM_003106	SRY (sex determining region Y)-box 2
*VIM*	NM_003380	Vimentin
*VSX2*	NM_182894	Visual system homeobox 2
*TJP1*	NM_175610	Tight junction protein 1 (zona occludens 1)
*OCLN*	NM_002538	Occludin
*CALB1*	NM_004929	Calbindin 1, 28kDa
*NES*	NM_006617	Nestin
*MAP2*	NM_002374	Microtubule-associated protein 2
*TUBB3*	NM_006086	Tubulin, beta 3
*POU4F2*	NM_004575	POU class 4 homeobox 2
*POU4F1*	NM_006237	POU class 4 homeobox 1
*NEUROD1*	NM_002500	Neurogenic differentiation 1
*MAP1A*	NM_002373	Microtubule-associated protein 1A
*SYP*	NM_003179	Synaptophysin
*PVALB*	NM_002854	Parvalbumin
*CALB2*	NM_001740	Calbindin 2
*RBFOX3*	NM_001082575	RNA binding protein, fox-1 homolog (C. elegans) 3
*SAG*	NM_000541	S-antigen; retina and pineal gland (arrestin)
*GFAP*	NM_002055	Glial fibrillary acidic protein
*MAP2*	NM_002374	Microtubule-associated protein 2
*PROX1*	NM_002763	Prospero homeobox 1
*OPN1MW*	NM_000513	Opsin 1 (cone pigments), medium-wave-sensitive
*OPN1SW*	NM_001708	Opsin 1 (cone pigments), short-wave-sensitive
*RHAG*	NM_000324	Rh-associated glycoprotein
*GNAT1*	NM_144499	Guanine nucleotide binding protein (G protein), alpha transducing activity polypeptide 1
*GNAT2*	NM_005272	Guanine nucleotide binding protein (G protein), alpha transducing activity polypeptide 2
*GNB1*	NM_002074	Guanine nucleotide binding protein (G protein), beta polypeptide 1
*GNB3*	NM_002075	Guanine nucleotide binding protein (G protein), beta polypeptide 3
*GNGT1*	NM_021955	Guanine nucleotide binding protein (G protein), gamma transducing activity polypeptide 1
*GNGT2*	NM_031498	Guanine nucleotide binding protein (G protein), gamma transducing activity polypeptide 2
*GRK1*	NM_002929	G protein-coupled receptor kinase 1
*CNGA3*	NM_001298	Cyclic nucleotide gated channel alpha 3
*CNGB1*	NM_001297	Cyclic nucleotide gated channel beta 1
*CNGB3*	NM_019098	Cyclic nucleotide gated channel beta 3
*GRK7*	NM_139209	G protein-coupled receptor kinase 7
*CNGA1*	NM_000087	Cyclic nucleotide gated channel alpha 1
*ARR3*	NM_004312	Arrestin 3, retinal (X-arrestin)
*RGS9*	NM_003835	Regulator of G-protein signaling 9
*GNB5*	NM_016194	Guanine nucleotide binding protein (G protein), beta 5
*RGS9BP*	NM_207391	Regulator of G protein signaling 9 binding protein
*GUCY2D*	NM_000180	Guanylate cyclase 2D, membrane (retina-specific)
*GUCY2F*	NM_001522	Guanylate cyclase 2F, retinal
*GUCA1A*	NM_000409	Guanylate cyclase activator 1A (retina)
*GUCA1C*	NM_005459	Guanylate cyclase activator 1C
*SLC24A1*	NM_004727	Solute carrier family 24 (sodium/potassium/calcium exchanger), member 1
*CTBP2*	NM_022802	C-terminal binding protein 2
*GAD2*	NM_000818	Glutamate decarboxylase 2 (pancreatic islets and brain, 65kDa)
*SLC1A3*	NM_004172	Solute carrier family 1 (glial high affinity glutamate transporter), member 3
*LHX1*	NM_005568	LIM homeobox 1
*SPDEF*	NM_012391	SAM pointed domain containing ets transcription factor
*MERTK*	NM_006343	C-mer proto-oncogene tyrosine kinase
*PRPH2*	NM_000322	Peripherin 2 (RD, slow)
*ARMS2*	NM_001099667	Age-related maculopathy susceptibility 2
*HTRA1*	NM_002775	HtrA serine peptidase 1
*PDE6B*	NM_000283	Phosphodiesterase 6B, cGMP-specific, rod, beta
*PLEKHA1*	NM_021622	Pleckstrin homology domain containing, family A (phosphoinositide binding specific) member 1
*HMCN1*	NM_031935	Hemicentin 1
*FBLN5*	NM_006329	Fibulin 5
*TULP1*	NM_003322	Tubby like protein 1
*CERKL*	NM_201548	Ceramide kinase-like
*GUCA1B*	NM_002098	Guanylate cyclase activator 1B (retina)
*THY1*	NM_006288	Thy-1 cell surface antigen
*ITGB1*	NM_002211	Integrin, beta 1 (fibronectin receptor, beta polypeptide, antigen CD29 includes MDF2, MSK12)
*CD44*	NM_000610	CD44 molecule (Indian blood group)
*ENG*	NM_000118	Endoglin
*NT5E*	NM_002526	5'-nucleotidase, ecto (CD73)
*RPL7*	NM_000971	Ribosomal protein L7
*HPRT1*	NM_000194	Hypoxanthine phosphoribosyltransferase 1
*RPL13A*	NM_012423	Ribosomal protein L13a
*GAPDH*	NM_002046	Glyceraldehyde-3-phosphate dehydrogenase
*ACTB*	NM_001101	Actin, beta

### Experimental Animals

For all experimental procedures, animals were treated in accordance with the NIH guidelines for the care and use of laboratory animals, the ARVO Statement for the Use of Animals in Ophthalmic and Vision Research, and under a protocol approved by the Institutional Animal Care and Use Committee (IACUC #2006-2698, and AUP-18-145). RCS nude rat transplant recipients were generated by crossing pigmented dystrophic RCS rats (RCS-p+) with Hsd:RH-Foxn1rnu (mutation in the foxn1 gene; no T cells) rats.^35^ Recipient rats have a mutant MerTK gene and a T-cell deficiency resulting in immunocompromised and retinal degenerate rats. To prevent infections of nude rats, rats were inspected daily, and cages changed under a laminar flow hood. Eyes of nude rats were cleaned every 2 weeks under isoflurane anesthesia.

### Retinal Sheet Preparation

Retinal organoids (differentiation days 30–70, average 47 days) were selected based on transparency and morphological criteria (a hollow spherical shape with a laminated structure) under a phase contrast and stereoscope. The structures were prepared into retinal rectangular sheets (0.7–1.3 × 0.6 mm) for transplantation. RPE “blobs” were dissected away. A second, smaller piece (0.2–0.3 × 0.6 mm) was also cut to be used as a “spacer” piece against the mandrel (to prevent the main piece from sticking to the mandrel).

### Transplantation

Recipient rats (P44-56, either sex) were randomized into age-matched nonsurgery (*n* = 13), sham (*n* = 16), and transplant (*n* = 33) cohorts. The group size for transplanted animals was set higher because some transplanted animals were used only for histology (*n* = 19) and not for functional tests to investigate transplant development. Two rats were eliminated after the first or second optical coherence tomography (OCT) examination because of faulty surgeries or corneal ulcers. Two rats could not be used for the final analysis because they died from anesthesia after OCT.

The animals were anesthetized with ketamine/xylazine (40–55 mg/kg Ket, 6–7.5 mg/kg Xyl), pupils dilated with 1% atropine eye drops (Akorn Pharmaceuticals, Lake Forest, IL). Before anesthesia, rats received a subcutaneous injection of ketoprofen (4 mg/kg) (Parsippany-Troy Hills, NJ) and dexamethasone eye drops (Bausch & Lomb Inc., Rancho Cucamonga, CA) to prevent eyelid swelling, and the eye was disinfected with ophthalmic betadine (Alcon, Fort Worth, TX). The nonsurgical eye was kept moist with application of artificial tears (Akorn). During the surgical procedure, the eye was frequently treated with 0.5% tetracaine (Bausch & Lomb) and 0.1% dexamethasone eye drops (Bausch & Lomb). Transplantation of retinal sheets has been previously described by our laboratory.^22^^,^^36^ Briefly, a small incision (approximately 1 mm) was made posterior to the pars plana, parallel to the limbus, followed by local retinal detachment. Retinal transplant tissues (one regular size and one small one as spacer, see above) were delivered to the subretinal space of the left eye using a custom implantation instrument. Sham surgery consisted of placing the instrument into the subretinal space and injecting media alone. The incision was closed with 10-0 sutures. For recovery, rats were given a subcutaneous injection of Ringer saline solution and the analgesic buprenorphine (Buprenex) (0.03 mg/kg) (Reckitt Benckiser Pharmaceuticals, Richmond, VA) for pain management. The surgical eye received additional treatment with betadine, followed by gentamycin/polymycin/bacitracin ointment (Bausch & Lomb). Rats were placed in a Thermocare (Thermocare, Paso Robles, CA) incubator for recovery.

### Spectral Domain OCT (SD-OCT) Imaging

SD-OCT imaging was used to document and monitor the transplant as it developed in the host retina. The general protocol was described previously.^22^^,^^36^ The SD-OCT images of the retina were obtained using a Bioptigen Envisu R2200 Spectral Domain Ophthalmic Imaging System (Bioptigen, Research Triangle Park, NC) after anesthesia with ketamine/xylazine and pupil dilation with atropine. Scans of a 2.6 × 2.6 mm area were taken to include the optic disk. If necessary, additional scans were taken to include areas in the further retinal periphery. The 488 × 488 × 5 (# B-scans/#A-scans/B-scan averaging value) scans were used to obtain fundus images; then the same area was scanned by 800 × 20 × 80 or 700 × 70 × 25 scans to obtain B-scans. Transplanted rats (*n* = 33) were imaged every 1 to 2 months, starting 2 weeks after surgery, up to 9.5 months of age (8.5 months after surgery). Rats with transplant misplacement into the vitreous or excessive surgical trauma such as optic nerve or corneal damage were excluded from further analysis after the first or second examination (*n* = 2). The last scan was scheduled as close as possible to the terminal experiment (superior colliculus [SC] recording). Sham (*n* = 10) and control nonsurgery RCS nude rats (*n* = 10) were imaged at approximately similar ages. Using 488 × 488 × 5 scans, B-scan images were used to outline the transplant edges on corresponding fundus images. Then Image-J was used to calculate the size (area) of the transplants. In B-scans, the transplant showed the same reflectivity as the host retina. B-scan images were carefully examined to exclude high reflections caused by scars.

### Electroretinography (ERG)

ERG was performed before surgery (age 4–5 weeks), and at 2, 4, and 6 months post surgery using the HMsERG system (Ocuscience, Las Vegas, NV) as previously described.^36^^,^^37^ Rats were dark-adapted overnight before testing, then anesthetized with ketamine/xylazine and 0%–1.5% isoflurane. Pupils were dilated using 1% atropine eye drops (Akorn Pharmaceuticals). Contact lens electrodes were placed on the cornea of both eyes, with reference and ground electrodes placed subcutaneously. An optically clear ophthalmic gel was used to maintain hydration and conductivity between the cornea and recording electrodes. Scotopic testing was conducted with flash stimuli intensities ranging from 1 to 25,000 millicandela followed by photopic testing (light adaptation of 10 minutes before the photopic test that records flash stimuli responses of 10–25,000 millicandela).

### Optokinetic Test (OKT)

At the age of 1.5, and 4 to 8 months, corresponding with 1 week before surgery and monthly at 2 to 6 months after surgery, the visual acuity of RCS nude rats (transplanted, sham surgery, and nonsurgery age matched controls) was measured by recording videos of optomotor responses to a virtual cylinder with alternating black and white vertical stripes (Optomotry, Cerebral Mechanics Inc., Alberta, Canada) as described previously.^22^^,^^36^ Some rats missed testing time points either because they were treated for infections or had been recorded in the SC.

Rats were dark-adapted for at least 1 hour before testing. Optomotor responses were recorded at 6 different spatial frequencies (0.05–0.45 cd/m^2^) for 1 minute per frequency by testers blinded to the experimental condition. Both the left and right eyes were tested by alternating the direction of the moving stripes. Two independent tests were performed at each time point, with at least 1 hour in between, with one test going from lowest to highest frequency, and the other from highest to lowest frequency. The best visual acuity of the two tests was used for analysis. All tests were video recorded and evaluated off-line by two independent observers blinded to the experimental conditions. Any discrepancies between the two observers resulted in a reanalysis of videos by a third observer, and data discussion before giving a final score, and before decoding the experimental condition.

### Superior Colliculus (SC) Electrophysiology

Responses to light flashes were recorded from the surface of the exposed SC as previously described.^22^^,^^36^ During recording, the tester was blinded to the group allocation of the animal. After overnight dark adaptation, responses from transplanted RCS nude rats (*n* = 14) were recorded between 5.9 and 9.7 months after surgery (age 7.5–11.3 months) and compared with responses from age-matched, nontransplanted RCS nude (*n* = 13), and sham rats (*n* = 16). A tungsten microelectrode (0.5 MΩ impedance; MicroProbe, Inc., Carlsbad, CA) was used to record multiunit electrical responses from 50 to 55 locations on the superficial layer of the SC, approximately 200 to 400 µm apart (ADInstruments, Inc. Colorado Springs, CO). Light stimuli (20 ms duration) were delivered approximately 10 times at 10-second intervals at an intensity of 0.58 to –6.13 log cd/m^2^. When responses were found, the intensity of the light stimuli was decreased until there was no response to determine the threshold. Responses to the strongest light stimuli (stimulus level 0.58 log cd/m^2^) were quantified and formed into a map over the area of the SC. Any up or down deflection higher than the background recording in the 100 ms before stimulation was considered a response (spike). All spikes occurring at 30 to 210 ms after the onset of the photic stimulus were counted. The sum was averaged across stimulus presentations. Analyses of the responses (spike counts and locations) were performed using a custom MATLAB program (Mathworks, Natick, MA).^13^^,^^22^

### Histology and Immunofluorescence

After injection of anesthetic overdose, rats were perfusion fixed with cold 4% paraformaldehyde in 0.1 M Na-phosphate buffer at 1.8 to 10.0 months after surgery (transplanted rats, *n* = 33; sham surgeries, *n* = 16; age-matched controls (AMC), *n* = 13). Eye cups were dissected along the dorsoventral axis, infiltrated overnight in 30% sucrose before embedding in Tissue-Tek O.C.T. compound and frozen using –60°C isopentane on dry ice. Retina organoids were frozen in O.C.T. using a similar procedure. Serial 10 µm cryostat sections were cut and stored at –20°C. Every fifth slide was stained using hematoxylin and eosin and analyzed for the presence of donor tissue in the subretinal space of the RCS host. Hematoxylin and eosin–stained slides were imaged on an Olympus BXH10 using an Infinity 3-1U camera. For immunofluorescence and diaminobenzidine (DAB) analyses, cryostat sections underwent antigen retrieval at 70°C with Histo-VT One (Nacalai USA Inc., San Diego, CA) and blocked for at least 30 minutes in 10% donkey serum (IFA) or 20% horse serum (DAB). Primary antibodies are listed in Table 2. Primaries were left on sections overnight at 4°C at specified concentrations. After several phosphate-buffered saline (PBS) washes, slides were incubated for at least 30 minutes at room temperature in fluorescent secondary antibodies, Alexa Fluor 488 donkey anti-rabbit IgG (H+L), Rhodamine X donkey anti-mouse IgG (H+L) and AF647 donkey anti-goat IgG (H+L) or biotinylated conjugated secondary antibodies (dilution of 1:200–1:400) (Jackson Immunoresearch, West Grove, PA). Fluorescent sections were coverslipped using Vectashield mounting media (Vector Labs, Burlingame, CA) with 5 µg/mL 4,6-diamidino-2-phenylindole. The Stem Cell 121 (SC121) or Ku80-stained sections were incubated with an ABC kit (Vector Labs, Burlingame, CA) and developed with DAB for up to 4 minutes and according to manufacturer's instructions. Fluorescence was imaged using a Zeiss LSM700 confocal microscope (Zeiss, Oberkochen, Germany) taking tiled stacks of 5 to 8 micron thickness at 40× (selected images). Zen 2012 software (Zeiss, Oberkochen, Germany) was used to extract confocal images. Three-dimensional images were extracted separately for each channel and combined in Adobe Photoshop CS6 software (San Jose, CA). Volocity (×64) software (Perkin-Elmer, Waltham, MA) was used to obtain higher magnification three-dimensional opacity rendered images that could be rotated for better viewing of three-dimensional structures including cell bodies and processes, in addition to co-localization analysis. In addition, Imaris software (Oxford Instruments) was used to analyze colocalization (see Fig. 7).

**Table 2.** Antibodies Used in this Study

Antigen	Species	Specific for	Dilution	Supplier	Catalogue #	RRID
Primary antibodies						
Brn3b	Rabbit	Ganglion cells (transcription factor POU4F2)	1:100 (fl.Ab)	Abcam (Eugene, OR)	ab56026	AB_880587
Chx10/Vsx2	Sheep	Retinal progenitors (transcription factor)	1:200 (fl.Ab)	Thermo Fisher (Huntington Beach, CA)	PA1-12565	AB_2216011
CRALBP	Rabbit	RPE and Muller cells	1:1K (fl. Ab)	Dr. John Saari (Univ. of WA)^60^	N/A	AB_2314227
CRALBP	Mouse	RPE and Muller cells	1:500 (fl. Ab)	Abcam	ab15051	AB_2269474
RLBP1(=CRALBP)	Rabbit	RPE and Muller cells	1:200 (fl. ab)	Fitzgerald (North Acton, MA)	70R-19908	N/A
CRX	Rabbit	Photoreceptor progenitor	1:200 (fl.Ab)	Santa Cruz Biotechnology,	sc-30150	AB_2276566
GFAP (Clone GA-5)	Mouse	Reactive glial cells	1:1K (fl. ab)	Sigma	G3893	AB_477010
GFAP (Clone GA-5)	Mouse	Reactive glial cells	1:1K (fl. ab)	Calbiochem/Millipore	IF03L	AB_212974
GFAP (Clone GA-5)	Mouse	Reactive glial cells	1:100 (fl. Ab)	Cell Signaling Technology (Danvers, MA)	#3670	AB_561049
Glutamine synthetase	Mouse	Müller cells	1:2K (fl. Ab)	Transduction labs/BD Biosciences, San Jose CA	610518	AB_397880
Iba1	Rabbit	Microglia	1:100 (fl. Ab)1:500 (ABC)	Biocare Medical (Pacheco, CA)	901-290-031218	N/A
Ku80	Rabbit	Human nuclei	1:400 (fl. Ab)1:2K (ABC)	Abcam	ab80592	AB_1603758
MAP2	Mouse	Ganglion cells	1:200 (fl.Ab)	Millipore	MAB3418	AB_94856
Otx2	Rabbit	Homeobox transcription factor in retinal and forebrain development	1:500 (fl.Ab)	Chemicon/Millipore	AB9566	AB_2157186
PKC α	Mouse	Rod bipolar cells	1:50 (fl. Ab)	Amersham (Little Chalfont, U.K.)	N/A (purchased 1990)	N/A
Recoverin	Rabbit	Photoreceptors, cone bipolar cells	1:2K (fl. Ab)1:10K (ABC)	Millipore	AB5585-I	AB_2253622
Red/green opsin	Rabbit	Red and Green cones	1:2K (fl. Ab)	Chemicon/Millipore	AB5405	AB_177456
Rhodopsin (rho1D4)	Mouse	Rods	1:100 (fl. Ab)1:10K (ABC)	Dr. Robert Molday, Univ. of British Columbia^61^	N/A	N/A
α-Synuclein; clone D37A6	Rabbit	Rodent amacrine cells and IPL; OLM (general)	1:100 (fl. Ab)	Cell Signaling Technology	4179	AB_1904156
Synaptophysin	Goat	Membrane protein of synaptic vesicles	1:100 (fl. Ab)	Novus Biologicals (Littleton, CO)	AF5555	N/A
SC-121 (STEM121)	Mouse	Cytoplasm of human cells	1:2K (fl. Ab)1:25K (ABC)	Stem Cell Inc. (Newark, CA)	AB-121-U-050	AB_2632385
Secondary antibodies						
Alexa Fluor 488	Donkey	Rabbit IgG (H+L)	1:400	Jackson Immuno Research (West Grove, PA)	711-545-152	AB_2313584
Rhodamine Red-X	Donkey	Rabbit IgG (H+L)	1:400	Jackson Immuno Research	711-295-152	AB_2340613
Alexa Fluor 647	Donkey	Rabbit IgG (H+L)	1:400	Jackson Immuno Research	711-605-152	AB_2492288
Biotin-SP	Donkey	Rabbit IgG (H+L)	1:200	Jackson Immuno Research	711-065-152	AB_2340593
Alexa Fluor 488	Donkey	Mouse IgG (H+L)	1:400	Jackson Immuno Research	715-545-150	AB_2340846
Rhodamine Red-X	Donkey	Mouse IgG (H+L)	1:400	Jackson Immuno Research	715-295-151	AB_2340832
Biotin-SP	Donkey	Mouse IgG (H+L)	1:200	Jackson Immuno Research	715-065-140	AB_2340783
Alexa Fluor 488	Donkey	Sheep IgG (H+L)	1:400	Jackson Immuno Research	713-545-147	AB_2340745
Rhodamine Red-X	Donkey	Sheep IgG (H+L)	1:400	Jackson Immuno Research	713-295-147	AB_2340737
Alexa Fluor 647	Donkey	Sheep IgG (H+L)	1:400	Jackson Immuno Research	713-605-003	AB_2340750
Biotin-SP	Donkey	Sheep IgG (H+L)	1:200	Jackson Immuno Research	713-065-003	AB_2340715
Biotin-SP	Donkey	Goat IgG (H+L)	1:200	Jackson Immuno Research	705-065-003	AB_2340396
Alexa Fluor 488	Donkey	Goat IgG (H+L)	1:400	Jackson Immuno Research	705-545-003	AB_2340428
Rhodamine Red-X	Donkey	Goat IgG (H+L)	1:400	Jackson Immuno Research	705-295-003	AB_2340422
Alexa Fluor 647	Donkey	Goat IgG (H+L)	1:400	Jackson Immuno Research	705-605-147	AB_2340437

CRALBP = cellular retinaldehyde binding protein, specific for Müller cells and RPE; GFAP = glial fibrillary acidic protein.

### Experimental Design and Statistical Analysis

Rats (either sex) were randomized into age-matched nonsurgery, sham, and transplant cohorts. The group sizes are similar to or exceed those reported in previous publications and that generally used in the field. Experimenters were blinded to the rat's experimental group. For all statistical analyses, the significance level was calculated in Graphpad Prism software (Graphpad Software LLC, La Jolla, CA) with paired and unpaired two-tailed *t* tests using mean ± SEM. The level of significance was set at 0.05.

### Code Availability Statement

The “hclust” R-program algorithm to analyze qPCR data was downloaded from The R Foundation and the code used to create the gene array scatterplots. The Matlab code that created the SC heatmaps from raw spike counts is a custom code. Both codes are available upon request.

## Results

### Development and Characterization of Retinal Organoids

**Figure 1.**
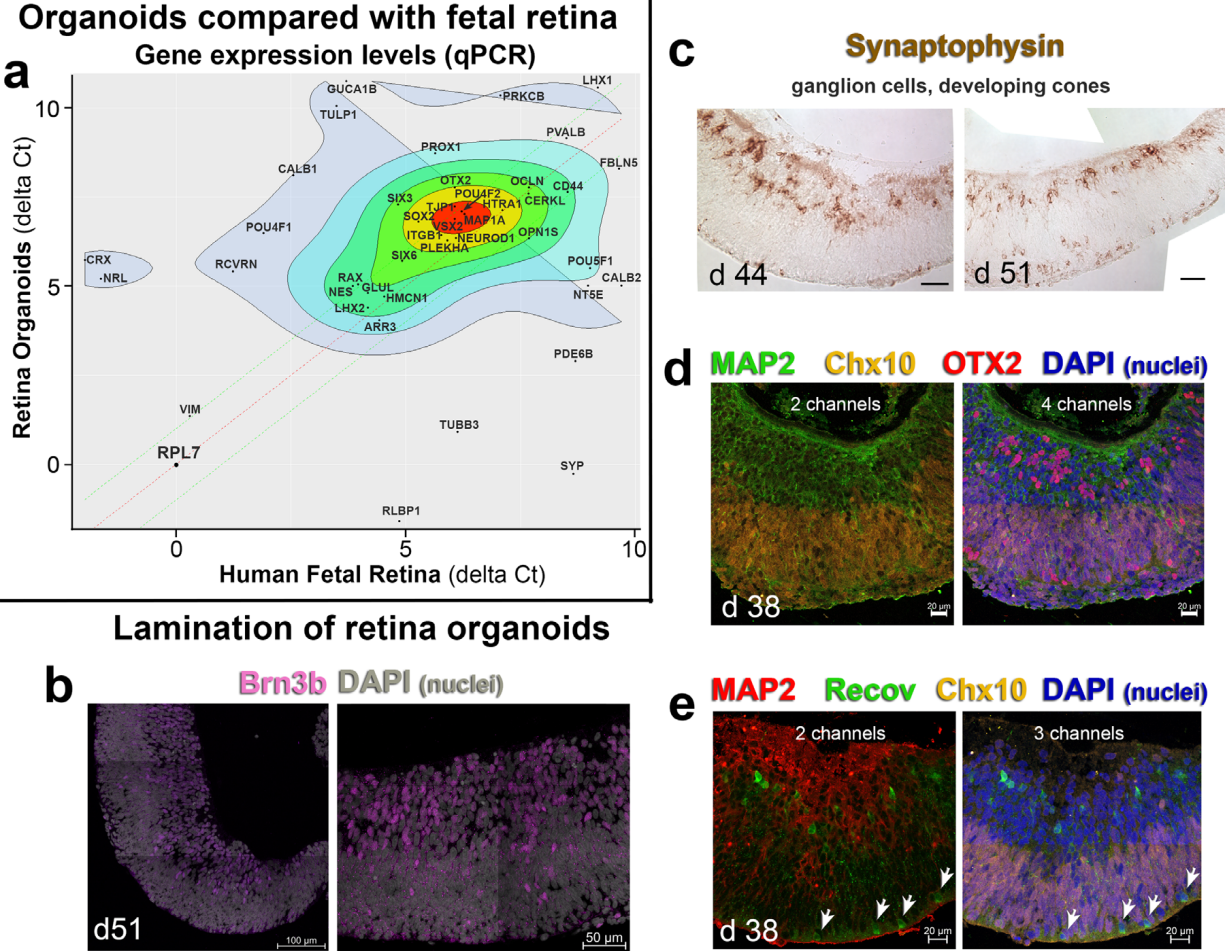
Characterization of retinal organoids. (**a**) Gene expression scatterplot (mean delta Ct) of d37-d70 retina organoids (*y*-axis) versus d105–d145 human fetal retina (*x*-axis). Highly co-expressed genes are shown in the center (*red*). The organoids (*n* = 17) and fetal tissue (*n* = 7) express similar levels of several important developmental genes. Differentially expressed genes are shown in the periphery (*grey*). The retina organoids contain all the major cellular subtypes responsible for support, synaptic integration, and phototransduction. These include glia, ganglion cells, bipolar, horizontal, amacrine, and Müller cells. *RPL7* was used as a housekeeping gene. The mean delta Ct values are calculated from biological replicates. (**b**) Confocal microscopy for retinal ganglion cell marker Brn3b (*magenta*) (D51). Nuclei are grey (DAPI). (**c**) Synaptophysin staining of d 44 and d 51 retina organoids, showing mostly developing retinal ganglion cells and cones. (**d**) Confocal microscopy of d38 retinal organoids for MAP 2 (*green*), Chx10 (*gold*), OTX2 (*red*), and DAPI (nuclei, *blue*). (**e**) Confocal microscopy of d38 organoids for MAP2 (*red*), Recoverin (*green*), Chx10 (*gold*), and DAPI (*blue*). The *white arrows* point to developing cone photoreceptors. Scale = 100 µm (**b**); 50 µm (**b**, **c**); 20 µm (**d**, **e**). DAPI = 4’,6-Diamidino-2-phenylindole.

CSC14 hESC developed into a refractive annular structure with a flattened area and a curved ridge of multilayered cells often surrounded by a RPE monolayer after 21 to 28 days of differentiation (d21–28) (modified procedure after^19^), as shown in our previous study.^22^ These structures were subsequently cut out between d30 and d70, and placed in suspension culture. Cut-out structures were designated as a “retinal organoid.” To confirm the success of our retinal organoids (developing into retinal progenitors), qPCR gene analysis (an array of 84 genes, see Table 1) was used to compare expression patterns between human fetal retina and stem cell derived retinal organoids (8 samples, d37–70 of differentiation). The to-be-transplanted retinal organoids acquired a gene expression pattern similar to human fetal retina (d105–145 of gestation) and contained all the important cell populations needed for successful integration within the host's microenvironment (Fig. 1a). Organoids showed lamination in their distribution of cell types. At d44 and d51, the ganglion cell marker Brn3b (Fig. 1b) and synaptophysin (a marker for presynaptic vesicles, Fig. 1c) was apparent in developing ganglion cells on the inner side of the organoids. Synaptophysin was also found in developing photoreceptor progenitors on the outside of the organoids (Fig. 1c). The localization of different retinal cell markers changed and could shift between apical and basal regions of the organoid during development. MAP2 (microtubule-associated protein 2) was found in putative ganglion cells and other neurons in the inner layer (Figs. 1d, 1e). CHX10 (VSX2, Visual System Homeobox 2) was found in retinal progenitor cells in the outer layer (Figs. 1d, 1e). OTX2 (Orthodenticle Homeobox 2, a transcription factor important for RPE, photoreceptor and bipolar cell development^38^) was found both in photoreceptor progenitors and in the putative inner retinal cell layers (Fig. 1d). Organoids also showed immunoreactivity to Recoverin (a marker for photoreceptors and cone bipolar cells), both in the developing outer nuclear layer and the inner retina (Fig. 1e) and CRX (data not shown). These findings suggest that with our “retinal determination” protocol, the CSC14 hESC differentiated into retinal organoids comparable with human fetal retina.

### In Vivo Development of Retinal Organoid Transplants Monitored by OCT

**Figure 2.**
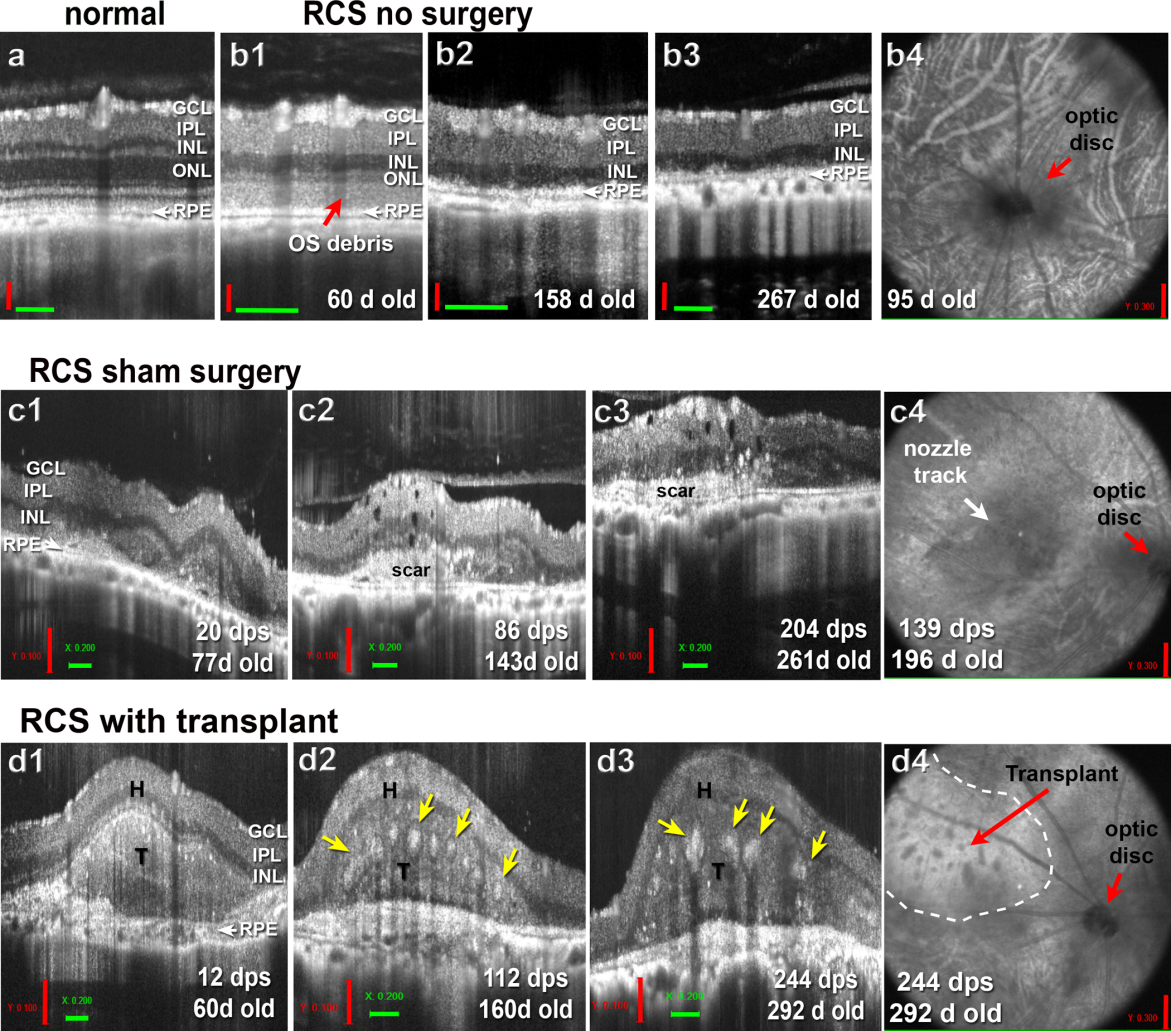
OCT analysis of transplant development and growth. (**a**) SD-OCT imaging of NIH-nude rat with normal retinal layers. (**b**) Age-matched control nude RCS rat. (**b1**–**b3**) B-scans show the RD between the ages of 60 to 292 days (2–10 months). At 60 days (**b1**), the outer segment debris can be seen in the subretinal space that is disappeared at the age of 158 days (**b2**). Note the thin retina. (**b4**) Example of a fundus image at 95 days of age. *Red arrow* points to optic disc. (**b**) Age-matched RCS sham surgery (injection of media only). (**b1**–**b3**) B-scans show scans through the nozzle track between the ages of 77 and 261 days (20–204 days post surgery [dps]). The white area in **c2** and **c3** indicates area of scar formation. (**c4**) Fundus image of this sham surgery rat at the age of 196 days (139 dps). (**d**) Example of SD-OCT imaging of transplant to nude RCS rat (transplant #3, see Table 3) at different time points, corresponding to the age-matched controls above: B-scans at 12, 112, and 244 dps (**d1**–**d3**). Photoreceptor rosettes (*yellow arrows*) can be seen around 4 and 8 months after transplantation (112 dps, 244 dps). (**d4**) In the fundus image, the transplant area can be identified as thicker retina (outlined with *white dashes*). Rosettes are seen as darker spots. GCL = ganglion cell layer; INL = inner nuclear layer; ONL = outer nuclear layer; T = transplant; H = host. Vertical scale bars (*red*) = 100 µm (**a**, **b1**–**b3**, **c1**–**c3**, **d1**–**d3**); = 300 µm (**b4**, **c4**, **d4**); horizontal scale bars (*green*) = 200 µm.

To monitor the development of retinal organoids after the transplantation, the rats were imaged with OCT monthly, starting at 2 weeks after transplantation. Figure 2a shows retinal layering by SD-OCT in the normal NIH nude retina. Age-matched control RCS rats showed reduced outer nuclear layer thickness in OCT imaging (Figs. 2b1–b4),^35^ similar to age-matched sham surgeries (example of sham surgery with nozzle trace in subretinal space shown in Figs. 2c1–c4). The transplants (average size 0.9 ± 0.03 mm^2^) were deposited subretinally and covered an average area of 3.6 mm^2^ at the last OCT scan (Table 3). Transplants continued to differentiate and developed photoreceptor rosettes several months post transplantation (Figs. 2d1–d4). These data show that retinal organoids could survive for a long time and mature after transplantation to nude RCS rats.

**Table 3.** SC Recording and OCT Analysis of Transplanted RD Rats

									Transplant Size (mm^2^)			
Rat ID	dps at SC Recording	Age (d)	Area with Response (%)	Max Spike Count (avg)	Response Threshold log (CD/m^2^)	Improved OKT @ 7 MPS (Y/N?)	Improved ERG @ 2 MPS (Y/N?)	Donor Age (Differentiation Day)	Surgery Day	Last OCT Scan	Fold Increase	dps at Last OCT Scan
Transplant 1	176	224	39.53	45.3	–1.01	Y	Y	38	0.86	3.30	2.84	110
Transplant 2	224	274	10.42	23	–0.15	Y	Y	52	0.92	1.06	0.15	207
Transplant 3	252	300	1.82	15.4	0.58	Y	N	39	0.91	1.50	0.65	244
Transplant 4	292	340	15.22	34.4	0.11	Y	N	39	0.96	4.84	4.04	287
Transplant 5	257	308	42.1	64.2	–1.91	Y	Y	51	1.1	5.96	4.42	244
Transplant 6	252	297	4.0	18.5	–0.15	N	N	41	0.92	6.56	6.13	216
Transplant 7	196	245	6.38	32.1	–0.41	N	N	59	1.01	3.02	1.99	187
Average	236 ± 13.9	284 ± 13.8	17.07 ± 5.88	33.27 ± 5.97	0.42 ± 0.29	5/7	3/7	45.6 ± 3.15	0.95 ± 0.03	3.75 ± 0.8	2.89 ± 0.81	214 ± 21.0
SC-recorded transplants without responses												
Transplant 8	217	270	n/a	n/a	n/a	Y	N	55	0.9	4.09	3.54	154
Transplant 9	222	272	n/a	n/a	n/a	N	N	55	0.93	3.38	2.64	77
Transplant 10	201	257	n/a	n/a	n/a	N	Y	45	0.75	3.58	3.77	89
Transplant 11	208	264	n/a	n/a	n/a	N	Y	45	0.86	4.54	4.28	203
Transplant 12	209	265	n/a	n/a	n/a	N	N	45	0.82	2.11	1.58	203
Transplant 13	245	289	n/a	n/a	n/a	Y	N	70	0.99	4.69	3.74	237
Transplant 14	229	284	n/a	n/a	n/a	N	N	30	0.72	1.93	1.68	224
Average	229.75 ± 5.22	281.5 ± 4.0				2/7	2/7	49.3 ± 4.7	0.85 ± 0.04	3.47 ± 0.42	3.03 ± 0.41	170 ± 24.4
Average (all)	227 ± 8.0	278 ± 7.66						47 ± 2.8	0.9 ± 0.03	3.61 ± 0.44	2.96 ± 0.44	192 ± 16.7

MPS = months after surgery.

### Analysis of Cell Type Development and Connectivity

**Figure 3.**
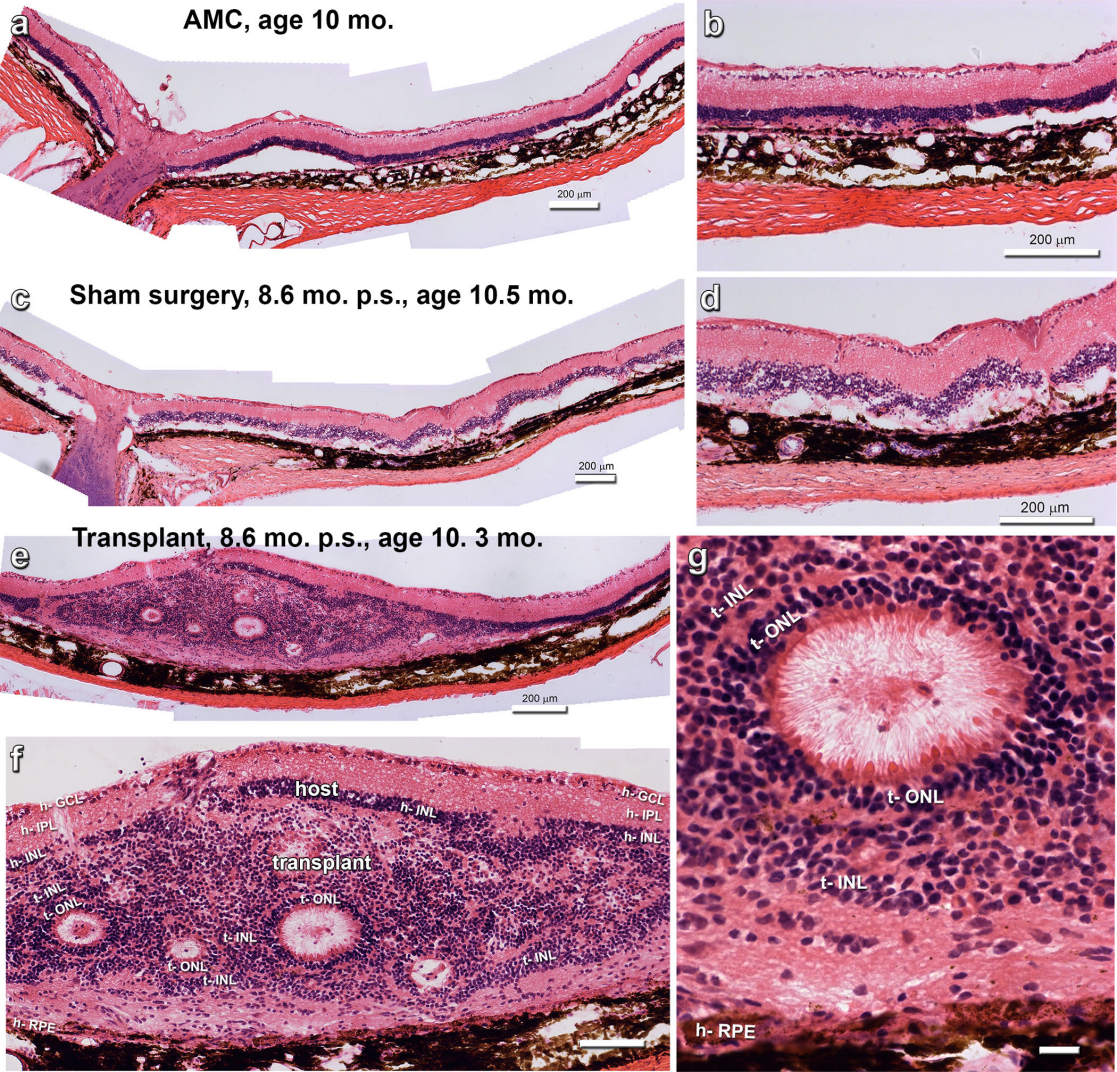
Representative hematoxylin-eosin images of experiments. (**a**, **b**) Example of a nonsurgery age-matched control (AMC), age 10 months. The photoreceptor layer has disappeared. (**c**, **d**) Representative example of sham surgery, 8.6 months after transplantation (p.s.), age 10.5 months. (**e**–**g**) Representative image of transplant #5. (**e**) Overview. (**f**) Enlargement. The transplants contains several photoreceptor rosettes. Also note that that the transplant appears to have penetrated the host inner nuclear layer in several areas. (**g**) Enlargement of photoreceptor rosette, with photoreceptor outer segments in center. Scale bars = 200 µm (**a**–**e**), 100 µm (**f**), and 20 µm (**g**). For layer abbreviations, see Table 5.

Figure 3 shows examples of hematoxylin-eosin stained sections of age-matched control (Figs. 3a, 3b), sham surgery (Figs. 3b, 3c) and transplant retina (Figs. 3e–g) at an age of approximately 300 days. Transplants developed their photoreceptors in rosettes (example in Fig. 3g) with inner and outer segments toward the rosette lumen.

### Bipolar Cells

**Figure 4.**
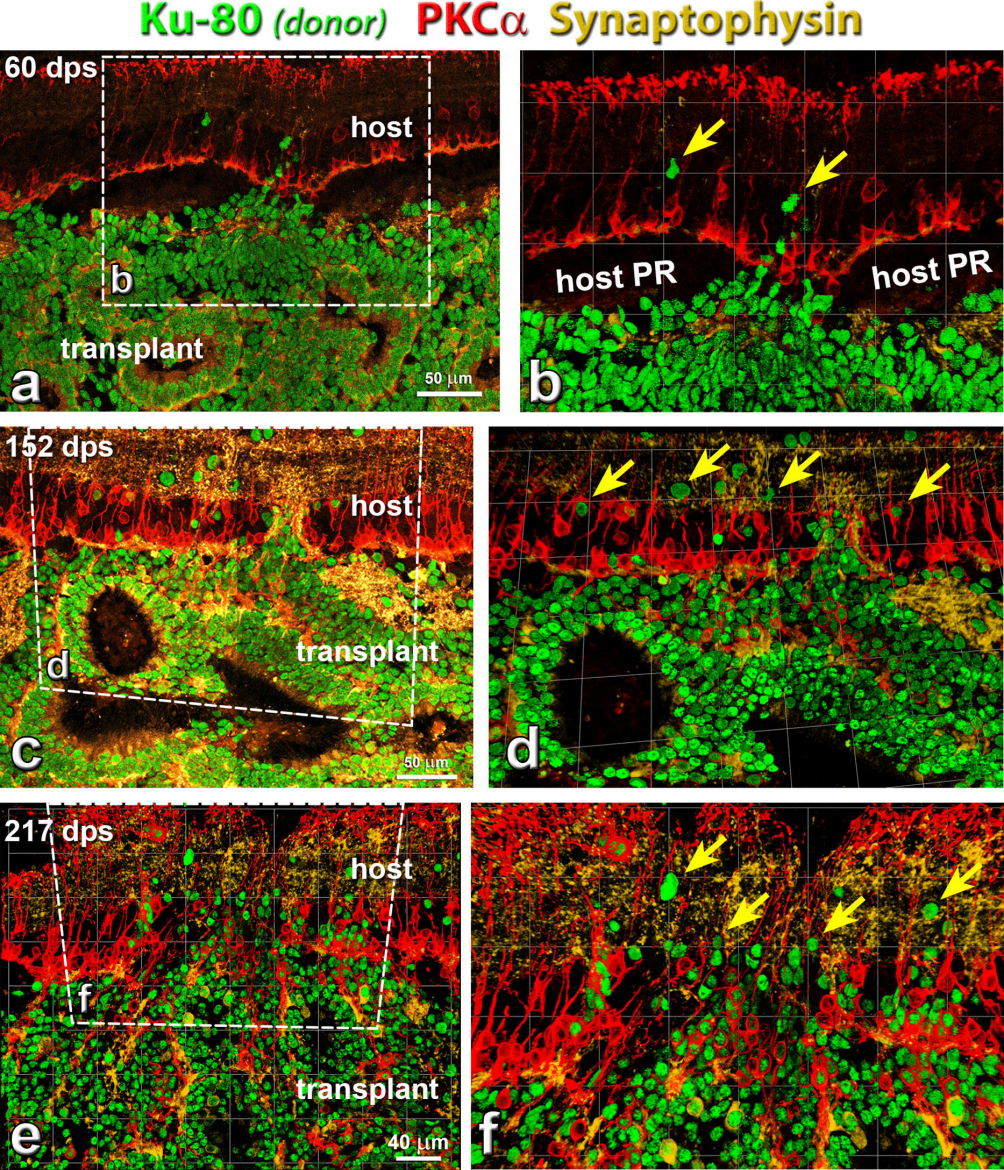
Rod bipolar cells (PKCα) development in transplant, and connectivity between host and donor cells at different time points. Combination of label for PKCα (protein kinase C α; rod bipolar cells, *red*), Ku-80 (human nuclei, *green*) and synaptophysin (membrane protein of human synaptic vesicles, gold). (**a**, **b**) Around 60 days post surgery (dps), transplants started to form rosettes. A few donor cells migrated into host retina (*yellow arrows*). PKCα and Synaptophysin staining was found in the transplant but not very strong. (**c**, **d**) Around 152 dps, more donor cells migrated into host retina (*yellow arrows*). PKCα and Synaptophysin staining increased in transplant. (**e**, **f**) At 217 dps, more donor cells migrated into host retina than at 152 dps (*yellow arrows*) (transplant #8, see Table 3). Extensive synaptic connectivity (synaptophysin) was found between donor and host cells in the host IPL. Scale bars = 50 µm. (**a**, **c**); 40 µm (**b**, **d**, **f**) are enlargement of (**a**, **c**, and **e**). For abbreviations, see Table 5.

The development of transplants (identified by Ku80, a marker for human nuclei) at different days after surgery (60, 152 and 217 days after surgery) is shown in Figure 4. PKCα-immunoreactive rod bipolar cells were found in both the host retina and transplants. Transplant PKCα immunoreactivity increased at later times after surgery (Figs. 4c–f), and transplant rod bipolar cells were oriented radially in the transplant inner nuclear layers. In some areas, host rod bipolar cells extended processes into transplants (Figs. 4d and 4f). In some transplants forming rosettes, transplant rod bipolar cells were very close to host rod bipolar cells and integrated with the host (Figs. 4d and 4f).

Transplant processes in the host inner plexiform layer (IPL) intermingled with host rod bipolar cell processes (Figs. 4d, 4f). Synaptophysin, a marker for presynaptic vesicles, exhibited strong staining in both host and transplant, indicating the good integration of host and donor tissues (Figs. 4c–f). Ku80 staining demonstrated that increasing numbers of donor cells migrated into and integrated with the host retina. Subsequently, the synaptophysin staining became stronger and the migration of donor cells and integration of host and donor cells became more extensive, indicating the continuing development of the donor tissues after transplantation.

### Photoreceptors

**Figure 5.**
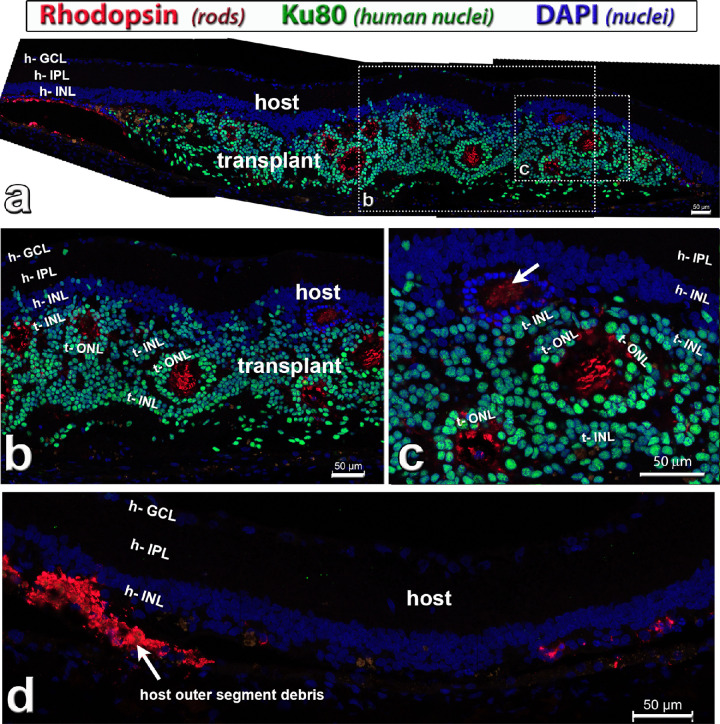
Rhodopsin staining (*red*) in hESC-retina transplant (*green nuclei*) to nude RCS rat, 9.7 months after surgery (see also Supplementary Fig. S1). This rat (transplant #4, see Table 3) had good responses in the SC. Combination of rhodopsin (rods, *red*), Ku80 (human nuclei, *green*) and DAPI (nuclei, *blue*). (**a**) Overview. The transplant has developed photoreceptors in rosettes with outer segments (*red*). There seems to be some host rod photoreceptor rescue close to the transplant (*left side* in **a**). Donor nuclei are migrating into the host retina in some places. (**b**, **c**) enlargements of (**a**). Note that there is also a rosette of remaining host photoreceptors (only blue DAPI stain) in (**c**) (*white arrow*). (**d**) Host retina outside transplant, with outer segment debris on the left, and few scattered degenerated host rod cell bodies on the right. Scale bars = 50 µm. DAPI = 4’,6-Diamidino-2-phenylindole. See Table 5 for definitions of labels.

**Figure 6.**
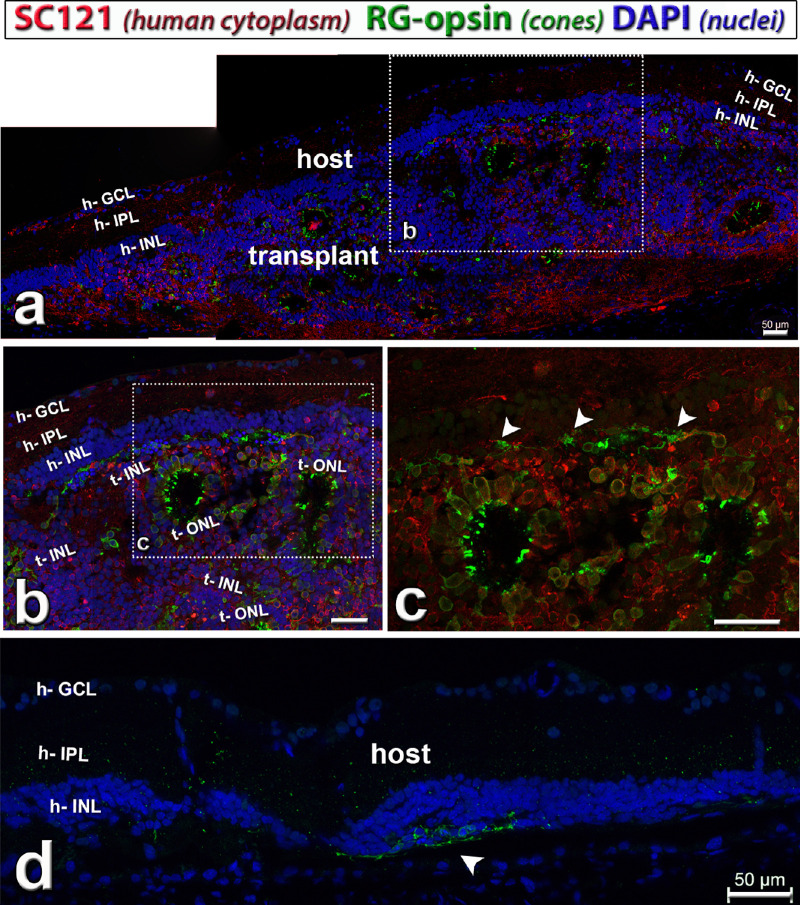
Cone photoreceptors (red–green [RG] opsin, *green*) in hESC-retina transplant (*red cytoplasmic label*) to nude RCS rat, 8.6 months after surgery (see also Supplementary Fig. S1). This rat (transplant #5) had good responses in the SC. Combination of SC121 (human cytoplasm, *red*), red–green opsin (cones, *green*) and DAPI (nuclei, *blue*). (**a**) Overview. The transplant developed photoreceptors in rosettes with outer segments (*green*). In the boxed area (enlarged in **b**, **c**) there were remaining host cone photoreceptors (*arrow heads* in **c**), but without outer segments. Donor processes in host IPL (**h**). (**d**) Host retina outside transplant depicting area with few degenerating host cones without outer segments (green, *arrow head*). Scale bars = 50 µm. DAPI = 4’,6-Diamidino-2-phenylindole. See Table 5 for definitions of labels.

Donor cells differentiated into photoreceptors after transplantation (Figs. 5, 6, and Supplementary Figure S1). Examples of rhodopsin-immunoreactive rod photoreceptors in the transplant and remaining photoreceptors in the host are shown in Figure 5 and Supplementary Figure S1c. In contrast with *Rho S334ter-3* RD nude rats, nude RCS rats retained their photoreceptors for a longer time. Supplementary Figure S1 shows still remaining host photoreceptor outer segments at 2 months after surgery (age 4 months) (Supplementary Fig. S1a–S1c), which had mostly disappeared at 5 months after surgery (age 7 months) (Supplementary Fig. S1d–S1f). At the age of SC recording (7.5–10.0 months), host photoreceptors had lost their outer segments (Figs. 5a [left], 5d). Rhodopsin staining showed that transplant rods extended outer segments into the rosette centers (Figs. 5a–c; Supplementary Fig. S1c, S1f).

Similarly, transplant cells developed into red–green opsin–positive cones (Figs. 6a–c; Supplementary Fig. S1a, S1d). Some host cones in the transplant area were rescued and survived for a longer time, but without outer segments (Figs. 6c, 6d; Supplementary Fig. S1a, S1b, S1d, S1e). Transplant cone outer segments (Figs. 6b, 6c; Supplementary Fig. S1d) seemed to be shorter than rod outer segments (Figs. 5b, 5c; Supplementary Fig. S1c, S1f).

### Synaptic Connectivity

**Figure 7.**
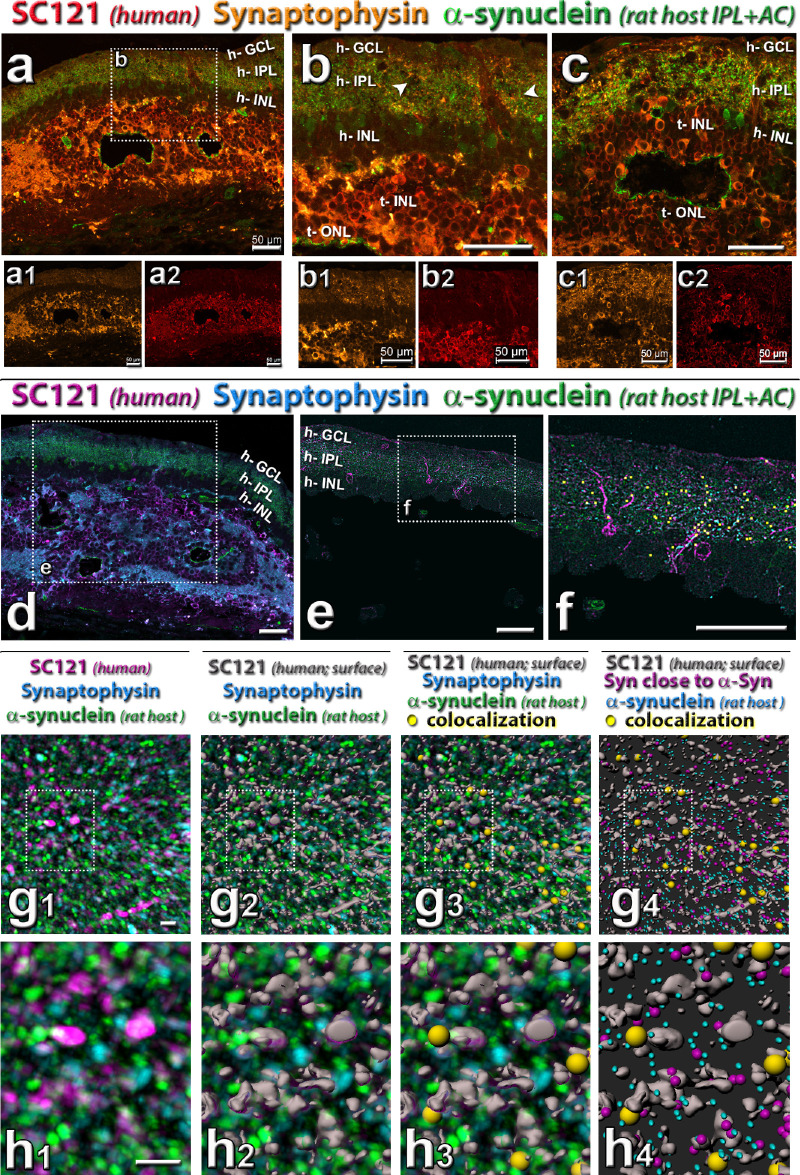
Triple staining for synaptophysin in combination with donor label SC121 and host cell marker (see also Supplementary Figs. S2 and S3). (**a**–**b**) Combination of SC121 (*red*), synaptophysin (*gold*), and rat-specific α-synuclein (*green*). Note the mixing of SC121, synaptophysin and α-synuclein in host IPL. (**a**, **b**, **d**–**h**) Same transplant as in Figure 5. *Arrowheads* in (**b**, **e**) point to areas of increased synaptophysin immunoreactivity and SC121 processes in the host IPL (h-IPL). (**c**) Same transplant as in Figure 6, showing transplant–host interface. (**d**) Combination of SC121 (*magenta*), synaptophysin (*turquoise*), and rat-specific α-synuclein (*green*): area adjacent to transplant shown in (**a**), with box showing area in (**e**). (**e**–**h**) Imaris software analysis of colocalization (closeness to) between host-specific α-synuclein, synaptophysin, and transplant label SC-121. (**e**) Mask applied to select mostly host retina. (f**)** Overlay of yellow squares shows colocalization of all three labels in the host IPL. (**g**, **h**) Enlarged three-dimensional rendering of detail in host IPL (all of the same area and magnification). The meaning of colors is indicated above the panels. (**g1**, **h1**) cleaned up label after thresholding. (**g2**, **h2**) SC121 staining rendered three-dimensional (*grey*) with Imaris surface function. (**g3**, **h3**) overlay of *yellow dots* showing triple colocalization. (**g4**, **h4**) Turquoise puncta indicate α-synuclein (host), purple puncta show synaptophysin close to α-synuclein, gray shows SC-121 surface; yellow dots show triple colocalization. Underlying image removed. Scale bars = 50 µm (**a**–**f**), 4 µm (**g**, **h**). See Table 5 for definitions of labels.

Transplant and host formed extensive synaptic connectivity as shown by antibodies specific for SC121 (human cytoplasm), synaptophysin (synaptic vesicles), in combination with recoverin (photoreceptors and cone bipolar cells) and rodent-specific α-synuclein (rodent IPL and amacrine cells) (Fig. 7; Supplementary Fig. S2), respectively. Recoverin immunoreactivity (Supplementary Fig. S2a–S2c) showed strong staining of transplant photoreceptors, whereas only scattered cones were found in the host. SC121 immunoreactivity showed transplant processes extending into the IPL of the host retina (Figs. 7b, 7b2; Supplementary Figs. S2, S3). Synaptophysin immunoreactivity in the host IPL was stronger close to the transplant than further away (Supplementary Fig. S3), suggesting intensive new synaptic connectivity between transplant and host (Figs. 7a, 7a1, 7b, 7b1). A rodent-specific α-synuclein antibody (Figs. 7a–7h4) demonstrated intermingling of transplant (SC121) and host (α-synuclein) processes in the host IPL, which also correlated with increased synaptophysin immunoreactivity (Figs. 7a1, 7b1, 7c1). Imaris image analysis of the host IPL with triple staining for SC121, Synaptophysin and rodent α-synuclein (Figs. 7d–7h4) demonstrated colocalization (“closeness”) of all three markers in the host IPL (indicated by yellow spheres in Figs. 7g3, 7g4, 7h3, 7h4). Overall, about 2% of rodent α-synuclein dots and 3% of synaptophysin immunoreactive dots in the host IPL were colocalized with SC121.

### Microglia

**Figure 8.**
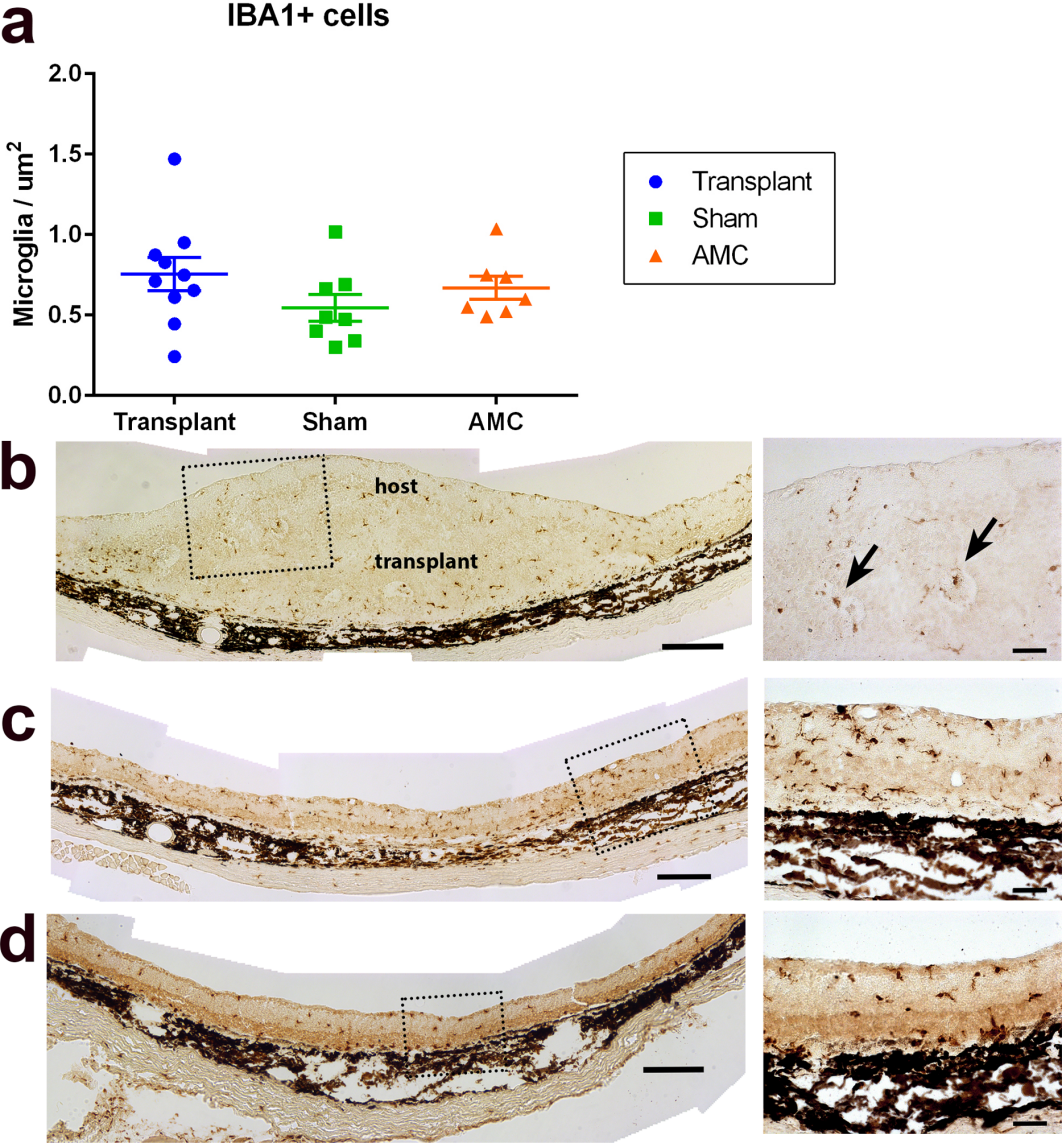
Host microglia cell response to RD and transplantation. (**a**) Count of Iba1-immunoreactivie cells (cells/µm^2^) in sections of transplants (*n* = 10), sham surgery (*n* = 8), and age-matched controls (AMC) (*n* = 7). The transplant group showed a slightly higher number of microglial cells than age-matched controls and sham groups, but the difference is not significant. (**b**) Iba1-staining in section of transplant #5 (257 dps, age 308 days). *Arrows* in enlargement point to microglial cells inside rosettes. (**c**) Representative section of sham surgery rat (210 dps, age 260 days). (**d**) Representative section of age-matched control (age 308 days). Scale bars = 200 µm (overviews); 50 µm (enlargements).

Iba1-immunoreactive microglia was observed in the retina of all three groups (age-matched controls, sham and transplant, Fig. 8). Microglia cells were also found within the transplanted tissue after transplantation. The number of Iba1 immunoreactive cells was higher in transplanted retinas (*n* = 10) than in sham (*n* = 8; *P* = 0.15) and age-matched control groups (*n* = 7; *P* = 0.56), but the above difference was not statistically significant (*P* > 0.05) (Fig. 8a). Interestingly, some Iba1+ microglia cells were observed in the center of the rosettes (Fig. 8b). Examples of microglial cells in sham surgery and age-matched controls are shown in Figures 8c and 8d, respectively.

### Müller Cells

**Figure 9.**
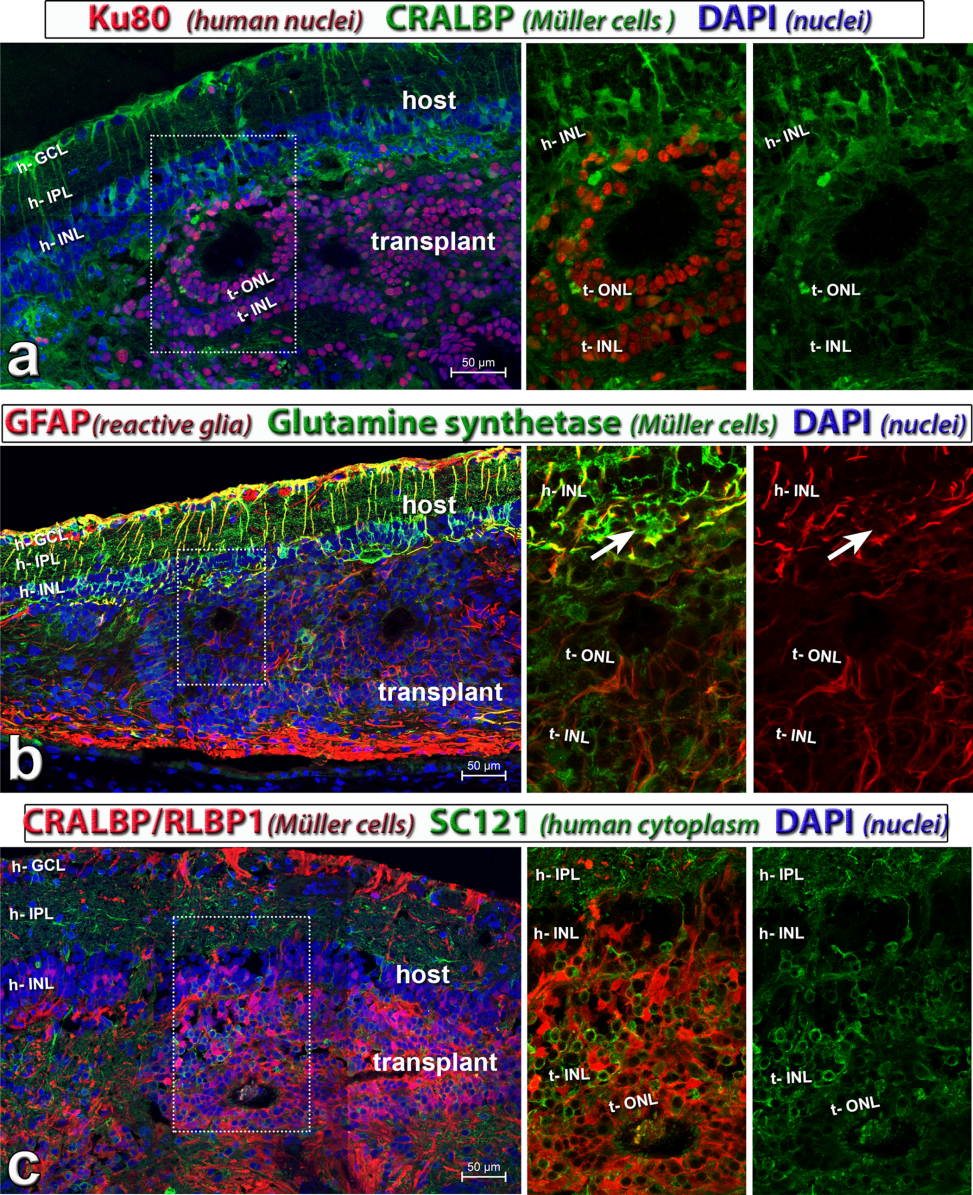
Glial markers (see also Supplementary Fig. S4). Transplant to RCS rat, 195 dps, age 245 days; donor tissue d59 of differentiation (transplant #7). This rat had a good response in the SC. The panels on the left show overview images. All nuclei are stained with DAPI (*blue*). Middle and right show enlargements of red and green channel, and images on the right show one channel only. (**a**) Combination of rabbit anti-Ku80 (human nuclei; *red*), and mouse monoclonal anti CRALBP (*green*). This monoclonal anti-CRALBP antibody stains the host stronger than the transplant**.** (**b**) Combination of GFAP (a marker for reactive glia; *red*), and glutamine synthetase (Müller cells; *green*). Müller cells in the host are more reactive than in transplant. The *white arrow* points to rosetted host photoreceptors. (**c**) Combination of polyclonal anti CRALBP/RLBP1 cellular retinaldehyde binding protein, Müller and RPE cell marker; *red*) and monoclonal anti-SC121 (human cytoplasm; *green*). The transplant has extended many processes into the host IPL. This polyclonal anti-CRALBP antibody stains the transplant much stronger than the host retina. Scale bars = 50 µm. CRALBP = cellular retinaldehyde binding protein, specific for Müller cells and RPE; DAPI = 4’,6-Diamidino-2-phenylindole; GFAP = glial fibrillary acidic protein. See Table 5 for definitions of labels.

Immunohistochemical staining for anti-cellular retinaldehyde binding protein, specific for Müller cells and RPE, glial fibrillary acidic protein (a marker for reactive glia) and glutamine synthetase (Müller cells) showed that the retina organoid transplants contained Müller cells that were radially oriented in rosettes (Figs. 9a–c; Supplementary Fig. S4). Müller cells in the host were more reactive than in the transplant as shown by glial fibrillary acidic protein staining (Fig. 9b). The transplants extended many processes into the host IPL that did not stain for cellular retinaldehyde binding protein, specific for Müller cells and RPE (Fig. 9c; Supplementary Fig. S4). RPE underneath the transplant appeared to be disrupted at times and generally expressed less RPE-specific markers, such as CALBP, Ezrin and RPE-65 than outside the transplant (data not shown). Nevertheless, such transplants could still elicit visual responses.

### Visual Function Improvement Evaluated by ERG

**Figure 10.**
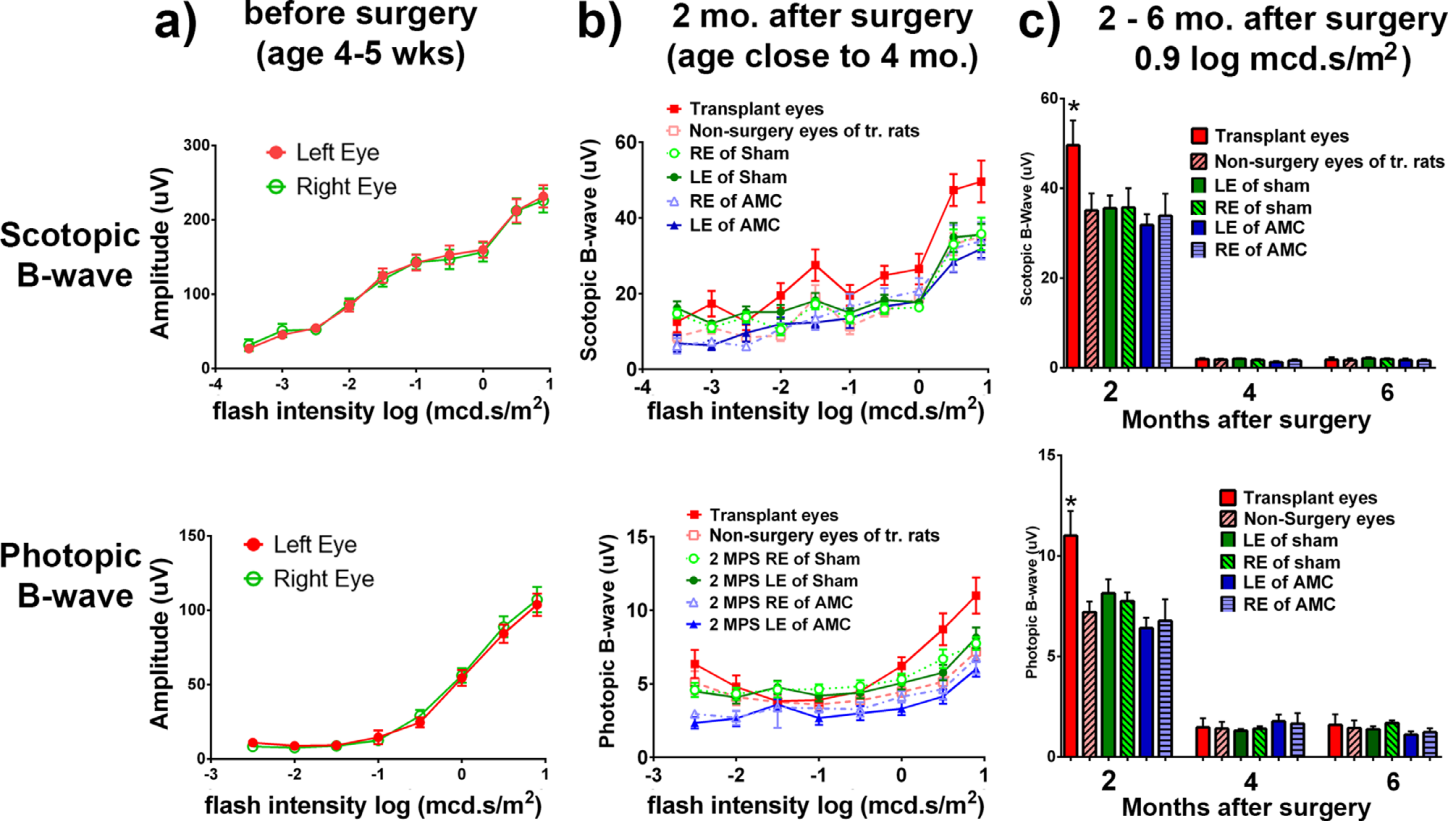
ERG recording results. (**a**) Before surgery: At 4 to 5 weeks of age, there were still measurable ERG scotopic and photopic B-waves which increased with light intensity. No difference was seen between the left and right eyes. (**b**) At 2 months after surgery (age around 4 months), the ERG responses have decreased significantly as the retina degenerated. However, eyes receiving transplants show significantly larger ERG scotopic and photopic B-waves than the nonsurgery control right eyes. (**c**) ERG decreases between 2 and 6 months after surgery. Although the transplanted eyes showed improvement at 2 months after surgery, ERGs were not detectable in both transplanted left eyes and nonsurgery control right eyes at later time points after surgery.

To determine whether the organoid could improve visual function, nude RCS rats were tested with ERG at 1 to 2 weeks before surgery (age 4–5 weeks) (Fig. 10a), and 2 to 6 months after transplantation (age 4–7 months) (Figs. 10b, 10c). Our data show at 2 months after surgery a significant improvement in ERG responses to scotopic (*P* = 0.038) and photopic stimuli (*P* = 0.008; *n* = 16) in transplanted eyes (left eyes) compared with nonsurgery eyes (right eyes); the improvement in scotopic ERG (transplant vs sham, *P* = 0.017; transplant vs AMC, *P* = 0.022) and photopic ERG (transplant vs sham, *P* = 0.036; transplant vs AMC, *P* = 0.009) is also significant when comparing transplanted eyes with that of sham and AMC (Figs. 10b, 10c). However, at later time points (4 and 6 months after surgery), ERGs were not detectable for all groups, and a similar difference was not observed (Fig. 10c). ERGs of age-matched control and sham surgeries decreased dramatically with age and showed no difference between both eyes. No ERG responses were detected in any of the RCS rats after 4 months after surgery (6- to 8-month-old RCS rats). These ERG data depicted that visual function improved by the retinal organoid transplants at 2 months after surgery.

### Visual Function Improvement Evaluated by OKT

**Figure 11.**
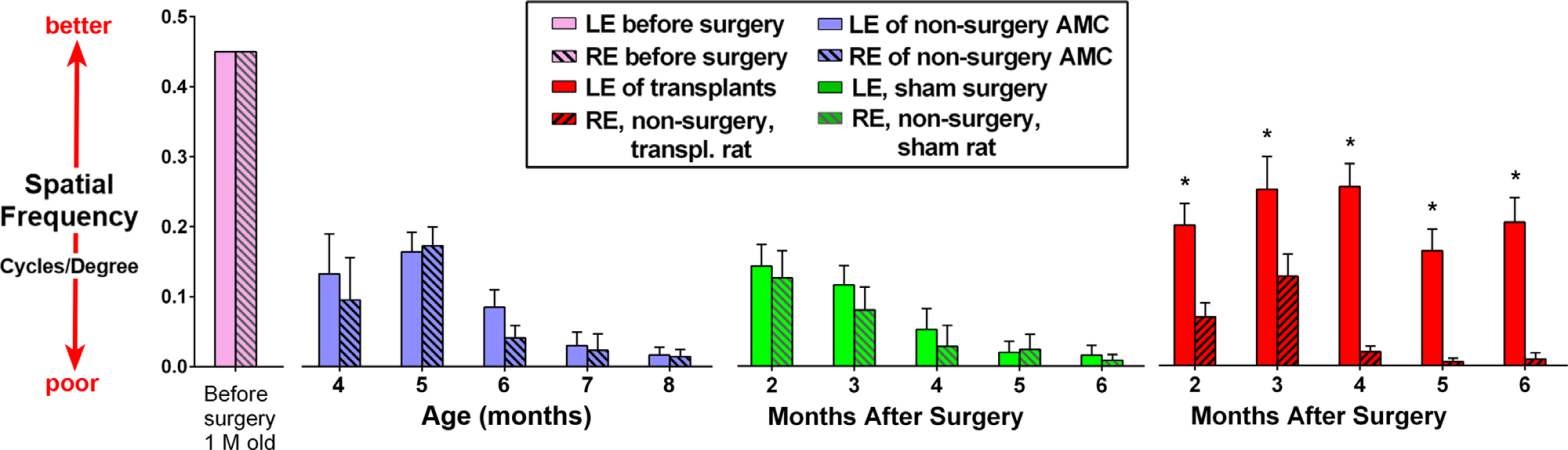
OKT results. OKT responses of right and left eyes were the same at the age of 1 month (approximately 1–2 weeks before surgery). For eyes of nonsurgery age matched controls and sham, and nonsurgery fellow eyes (right eyes) of transplant group, OKT responses significantly decreased with age. The transplanted eyes showed significant improvements compared with controls which were maintained up to 6 months after surgery, corresponding to the age of 8 months.

OKT of nude RCS rats showed that the visual acuity of age-matched RCS controls and sham RCS rats significantly decreased after 4 months after surgery (corresponding with an age of 6 months). In contrast, in the transplanted RCS rats, the visual acuity of eyes with hESC-derived retinal organoid transplants showed significant improvement from 2 (transplant *n* = 16, sham *n* = 6, AMC *n* = 5), 3 (transplant *n* = 15, sham *n* = 12, AMC *n* = 10), 4 (transplant *n* = 25, sham *n* = 6, AMC *n* = 10), 5 (transplant *n* = 15, sham *n* = 6, AMC *n* = 8), and 6 (transplant *n* = 13, sham *n* = 6, AMC *n* = 12), months after surgery. There was no difference between age-matched controls and sham control groups in visual acuity (*P* = 0.865, unpaired *t* test). In addition, there was no significant difference between the left eye and right eye of sham surgery and age-matched control rats (*P* = 0.735, paired *t* test). The nonsurgery eyes of the transplanted rats also showed a similar trend in visual acuity loss. However, a significant improvement in the visual acuity was observed in transplanted eyes compared with nontransplanted eyes (paired *t* test). This difference was more apparent at later time points (*P* = 0.001, *P* = 0.037, *P* < 0.0001, *P* < 0.0001, and *P* < 0.0001 at 2, 3, 4, 5, and 6 months after surgery, respectively; Fig. 11). Furthermore, the visual acuity of the transplanted eyes was significantly higher compared with the nonsurgery control and sham surgery rats (transplant vs sham, *P* = 0.01; transplant vs AMC, *P* = 0.007). All these values were lower than those before surgery (1 month old) as compared with normal (nondegenerate) NIH or left eye nude rats with a normal retina (0.42 ± 0.03 cycles/degree).^36^

### Visual Function Improvement Evaluated by SC Electrophysiologic Recording

**Figure 12.**
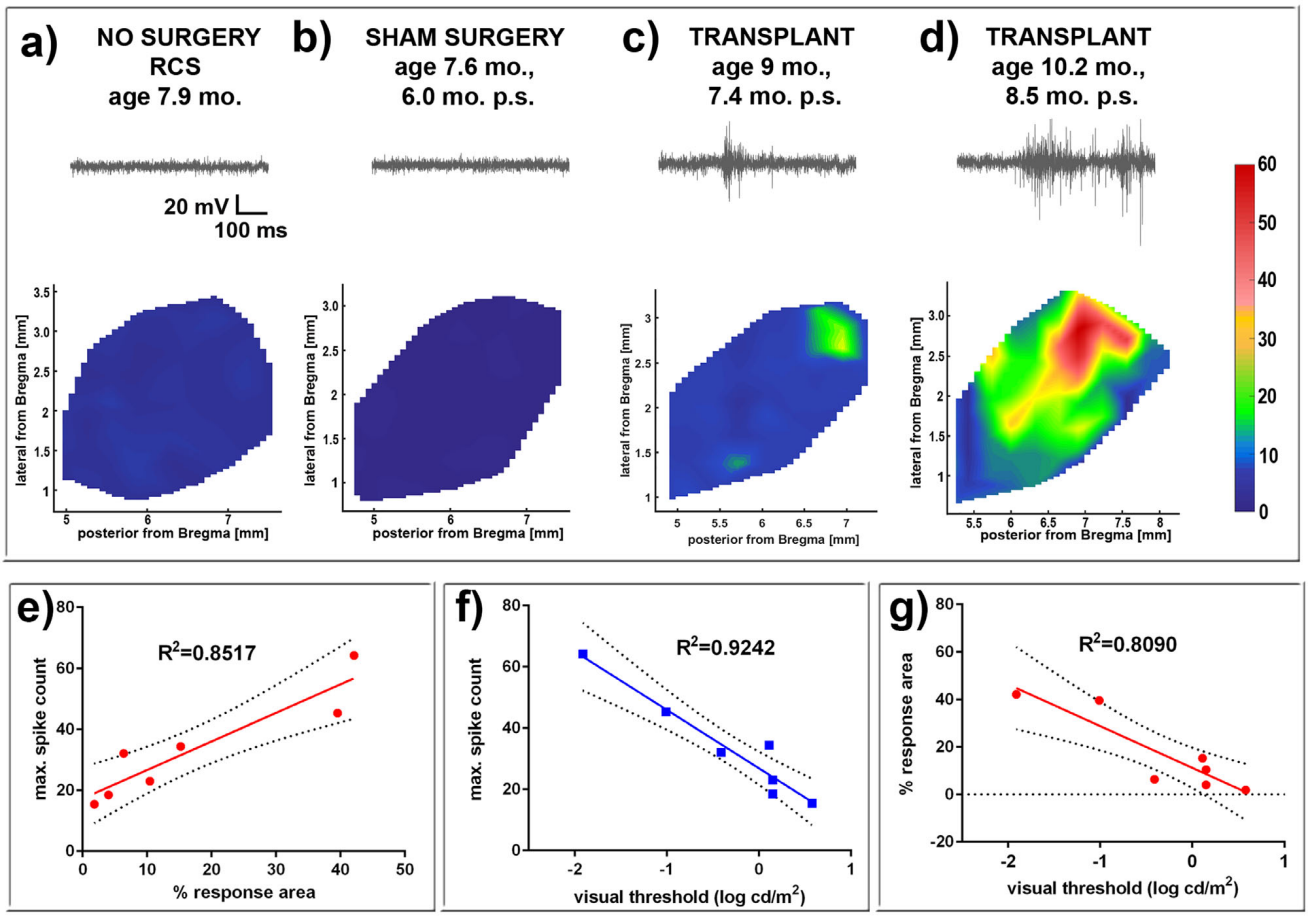
SC recording (see also Supplementary Fig. S5). Representative spike count heatmaps and sample traces of SC recorded visually evoked responses of (**a**) nonsurgery age matched control, (**b**) sham surgery, and two transplanted rats with responses (**c, d**; transplant #2 and #5). The sample traces start about 100 ms before the 20 msec light stimulus. No response was found in the entire SC area of nonsurgery age-matched control and sham rats. Strong responses were found in some areas of the SC in 7 of 14 recorded transplant rats (see Tables 2 and 3). (**e**) Maximum spike count versus percent response area (R^2^ = 0.8517). (**f**) Maximum spike count versus visual threshold (R^2^ = 0.9242). (**g**) Percent response area versus visual threshold (R^2^ = 0.8090).

Electrophysiologic recording in the SC was also used to test the visual function in the rats (Fig. 12; Supplementary Fig. S5). At the age of transplantation (1.5 months), there was still some residual visual activity in a small area of the SC which was disappeared at the age of 3 to 5 months in nonsurgery RCS rats (Supplementary Fig. S5, Table 4^5^). Strong visual responses (spike activity) were recorded in the SC from transplanted RCS rats (*n* = 7/14) (Figs. 12c, 12d) at the age of 7.5 to 11.3 months (5.9–9.7 months after surgery). Some of these rats (*n* = 2) even showed responses to very low levels of light stimulation at the scotopic level; the best light threshold was –1.91 log cd/m^2^ (Table 4). No responses could be recorded in the SC of control nonsurgery age-matched control rats (*n* = 13) and sham surgery rats (*n* = 16) (Figs. 12a, 12b) even at the strongest light stimulation tested (+0.58 log cd/m^2^). SC electrophysiologic activity (percentage of area with response, max spike count, and response threshold) in response to light of the transplanted rats was significantly higher compared with age-matched control and sham surgery rats (*P* = 0.0006; Tables 3 and 4). Further statistical tests (R^2^) in the seven transplant rats with responses to light showed that max spike count, response threshold and percentage of area with response strongly correlated with each other (Figs. 12e–12g). Five of the seven transplanted rats with responses in the SC showed improvements in OKT responses at the last test, whereas only three of the seven rats with responses in the SC showed ERG improvements at 2 months after surgery (Table 3). This finding demonstrates that retinal organoid transplants contribute to the visual functional improvement seen in the nude RCS rats.

**Table 4.** Summary of SC Electrophysiology Recording of All Rats

Experimental Group	Area With Response (%)	Max Spike Count	Response Threshold Log (CD/m^2^)	Age at Recording (Months)	Months After Surgery	No. of Rats With Response	Total Number (*N*)
1.5-month-old RCS rat	18.44 ± 5.40	45.87 ± 6.94	–0.43 ± 0.27	1.5	n/a	3	3
4-month-old RCS rat	0.00	0.00	n/a	4	n/a	0	4
5-month-old RCS rat	0.00	0.00	n/a	5	n/a	0	3
AMC Nonsurgery	0.00	0.00	n/a	5–11	n/a	0	13
Sham	0.00	0.00	n/a	5–11	4–10	0	16
Transplant to RCS rats	17.07 ± 6.36	33.27 ± 6.45	–0.42 ± 0.31	7.5–11.3	5.9–9.7	7	14
Transplant to *RhoS334ter-3* RD rats^22^	8.73 ± 3.44	25.8 ± 5.45	0.32 ± 0.09	5–11	4–10	9	13

**Table 5.** List of Abbreviations

Abbreviation	Expansion
2D	Two-dimensional
AMC	Age-matched control
AMD	Age-related macular degeneration
cDNA	Complimentary deoxyribonucleic acid
d	day
DAB	3-3’-Diaminobenzidine
DAPI	4’,6-Diamidino-2-phenylindole
dps	Days post-surgery
GCL	Ganglion cell layer
hESC	Human embryonic stem cell(s)
IHC	Immunohistochemistry
h-	Host
INL	Inner nuclear layer
IPL	Inner plexiform layer
iPSC	Induced pluripotent stem cell
OCT	Optical coherence tomography
OKT	Optokinetic test
ON	Optic nerve
OPL	Outer plexiform layer
P30	Postnatal day 30
PBS	Phosphate-buffered saline
PR	Photoreceptor
qPCR	Quantitative polymerase chain reaction
RCS	Royal College of Surgeons
RD	Retinal degenerate
RP	Retinitis pigmentosa
RPE	Retinal pigment epithelium
RT	Reverse transcriptase
SC	Superior colliculus
SC121	Stem cell 121 antibody
SD	Spectral domain
t-	Transplant
VA	Visual acuity

## Discussion

In the current investigation, consistent with a previous report,^22^ we demonstrate that hESC–retina organoid sheets exhibit high expression levels of several key genes for eye-field development, such as RAX, LHX2, Six3, Six6, and Chx10. The similarity between retina organoids and human fetal retina demonstrated the immature development of retinal progenitors in the retina organoids and its potential use as a therapeutic candidate. The present report demonstrates that after the transplantation into the RCS nude rats at advanced disease stage, retina organoid sheets survive and develop into mature photoreceptors and other retinal cells. Visual function tests (ERG, OKT, and SC electrophysiology) show visual improvement after transplantation.

For the functional photoreceptors, recycling of visual pigments by isomerization and oxidation, from all-trans retinol to 11-cis retinol, and to 11-cis retinal, is essential.^39^ The first essential step of isomerization to form 11-cis retinol is catalyzed by RPE65 in RPE for rod visual pigments (review^40^) and in Müller cells for cone visual pigments.^41^^,^^42^ Further oxidation is required to restore 11-cis retinal, which is then catalyzed by retinol dehydrogenases, distributed in the retina and RPE in a number of isoforms with overlapping activities.^39^^,^^43^^,^^44^ Another function of RPE is phagocytosis. Photoreceptor cells continuously generate new outer segments from their base while simultaneously releasing outer segments to be phagocytosed by the RPE monolayer.^45^^,^^46^

In the RCS rat, the genetic mutation in the MerTK gene causes defective phagocytosis of RPE, resulting in photoreceptor degeneration. Theoretically, replacement of both RPE and photoreceptors is needed to restore vision in RCS rats. However, our data show that retinal organoids transplanted without RPE could improve vision. These findings support the theory that RPE phagocytosis is not needed for vision improvement by the retinal organoid transplant; and that other cells, such as Müller cells^42^^,^^47^^–^^49^ and microglia might compensate for the RPE and provide the support required by photoreceptors.^50^ Interestingly, some Iba1+ microglia were found in the center of rosettes (location of photoreceptor outer segments) similar to what has been observed in fetal rat and hESC-retina organoid transplants (unpublished observations). The number of Iba1+ microglia was not significantly increased in the transplant group compared with sham and age-matched control groups, suggesting that the surgery did not cause an inflammatory response. The presence of Iba1+ microglia cells and Müller cells in the transplants may compensate for the lost phagocytic function of the RPE.^42^^,^^51^ However, we do need to be aware that RCS rat is a model of RPE dysfunction (loss of phagocytosis) instead of an RPE degeneration model. Phagocytosis is one of the main functions of RPE. Without it, photoreceptors will die. A recent study showed that surgical removal of debris in subretinal space of RCS rat could rescue the remaining photoreceptors.^52^ Further studies are required to test if the photoreceptors in the transplants produce fewer outer segments or if their outer segments are removed by other cells. Another possibility may be that stem cell-derived photoreceptors may have better capability to survive.

Our previous work^22^ showed that similar retina organoid (photoreceptor progenitor) sheet transplants improved vision in the RD *Rho S334ter-3* nude rats. Because the *Rho S334ter-3* rat loses all photoreceptors but still has functional RPE, it is understandable that the transplant could replace damaged photoreceptors and improve visual function. Our current finding that retina organoid sheet transplants also improved vision in the RCS nude rats suggests that transplants can function in the presence of dysfunctional RPE. However, the success rate of visual responses in the SC found in the RD nude rats (9/13) after transplantation was higher than that observed in the RCS nude rats (7/14). Although Müller cells or microglia can support photoreceptors, they may not compensate for the quality of support provided by RPE cells. This finding suggests that cotransplanting retina organoid and RPE may be desirable for a more robust restoration of the visual activity in retinal diseases caused by RPE dysfunction or degeneration.

After transplantation of fluorescently labeled dissociated cells, several studies have demonstrated the possibility of cytoplasmic material exchange between the transplanted cells and the host photoreceptors, instead of direct integration of transplanted photoreceptor precursors. This causes the rescue of host photoreceptors.^53^^–^^58^ Interestingly, only photoreceptor–photoreceptor or Müller–photoreceptor interactions showed the exchange of cytoplasmic material. The donor cells do not exchange intracellular content with other cells (horizontal, bipolar, Müller, or amacrine cells). Furthermore, the cytoplasmic exchange occurred only when the host outer limiting membrane was broken and an outer nuclear layer was present. In our study, there was still a thin outer nuclear layer present at the time of transplantation. Thus, there could be more cytoplasmic transfer in the RCS rat than in the *Rho S334ter-3* rat. However, Ku80 staining (a donor cell nuclear marker) also confirmed that donor cells migrated into and integrated with the host retina, and that the photoreceptors in the body of the transplant are of donor origin. Therefore, our data suggest that both integration and cytoplasmic transfer of donor cells with the host retina occurred in the current condition, and result in visual improvement.

After transplantation, the donor tissues survived for a long time (more than 9 months). The donor cells continued to grow and developed into mature retinal cells, including rod and cone photoreceptors, bipolar, Müller, horizontal, and amacrine cells, except ganglion cells. The human cytoplasmic marker SC121 demonstrated donor cell processes’ extension into the host retina. Extensive synaptic connectivity was found inside donor tissue and between donor and host cells, indicating synaptic integration between donor and host tissue that might contribute to the observed functional improvement. Interestingly, only a limited area of the SC showed responses to light stimuli after the transplantation, which corresponded with the small area of the host retina containing the transplant. Based on these data, it may be suggested that the transplant could improve vision by replacing the photoreceptors. However, the presence of some surviving host photoreceptors found near transplants in the RCS host retina is suggestive that the transplants also induced some rescue effect by release of neurotrophic factors.

Our present investigation showed significantly improved ERG responses to scotopic and photopic stimuli in transplanted eyes compared with controls at 2 months after surgery, but the ERG responses were not detectable in all groups at later timepoints. At 2 months after surgery, ERG was still detectable in all groups. At this time point, the transplant was not mature enough to replace the function of host photoreceptors; thus, the improvement in transplanted rats seems to be a trophic effect. This is also indicated by the fact that only three of the seven rats with SC responses showed improved ERG responses. Full-field ERGs give an overall assessment of the visual response of the retina, but are not sensitive enough in most cases to detect visual function elicited from a relatively small part of the retina.^59^ Because rescued photoreceptors are limited to the transplant area (up to 3.8 mm^2^ after full development), corresponding with less than 10% of the retina, conventional full-field ERG may not be capable of identifying visual responses elicited by the transplants alone. In similar experiments (data not shown), we have observed that SC responses but not ERG are recordable in 1- to 3-month-old RD *Rho S334ter-3* nude rats, confirming that SC recording is more sensitive than full-field ERG (see also^36^). Recording from a retinal wholemount in vitro would be sensitive enough to detect responses,^21^ but that recording would have required sacrificing the animal. Other possibilities are multifocal or focal ERGs; however, that process requires a different setup.

OKT data showed a significant decrease after 4 months after surgery (age 6 months) in sham and age-matched control RCS rats, whereas the transplants significantly improved visual acuity from 2 to 4 months after surgery to the end of study period (up to 6 months after surgery). Compared with our previous work in RD *Rho S334ter-3* nude rats,^22^ the vision improvement owing to retinal organoid transplants was more prominent in the current RCS rat study. This finding may be due to an additional rescue effect on host photoreceptors in combination with photoreceptor replacement. In contrast, almost all host photoreceptors were lost at the time of transplantation in the RD *Rho S334ter-3* nude rats.

In summary, our findings demonstrate that retinal progenitor sheets derived from hESC transplanted at an advanced disease stage improve the visual function in nude RCS rats by both cell replacement and cell rescue. This study provides proof of concept that retinal progenitor sheet transplantation may become a possible future treatment not only for RD but also for RPE dysfunction/degeneration diseases. RPE and retinal organoid (mainly photoreceptor progenitors) co-grafting may possibly result in better vision restoration in RD patients.

## Supplementary Material

Supplement 1

Supplement 2

Supplement 3

Supplement 4

Supplement 5
