# Supplementary material for: Retina Organoid Transplants Develop Photoreceptors and Improve Visual Function in RCS Rats With RPE Dysfunction
Source: Invest Ophthalmol Vis Sci. 2020 Sep 18;61(11):34. doi: 10.1167/iovs.61.11.34 (PMC7509771; doi:10.1167/iovs.61.11.34)
Supplement: Supplement 1 [file iovs-61-11-34_s001.pdf]

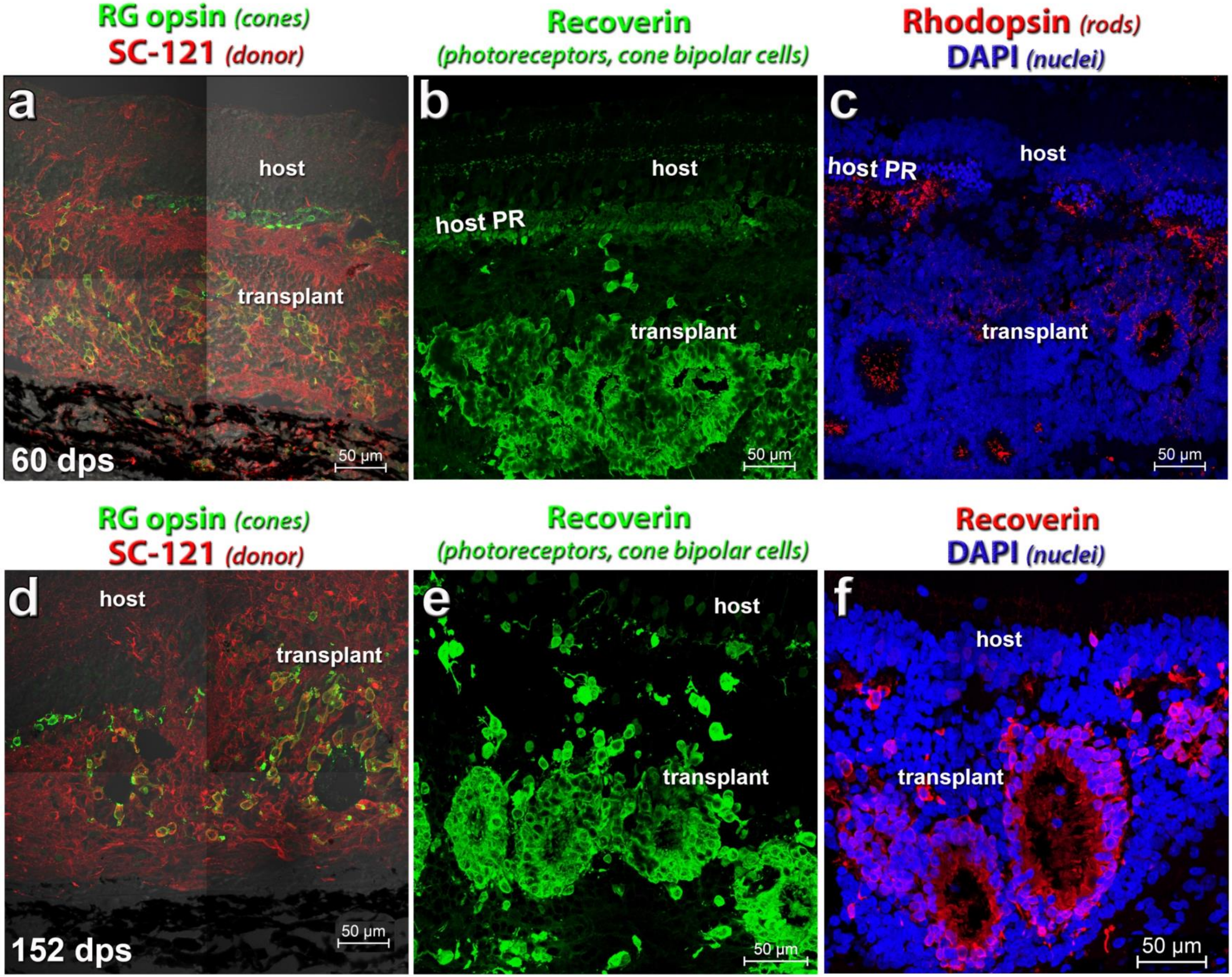
RG opsin (cones)
SC-121 (donor)
Recoverin
(photoreceptors, cone bipolar cells)
Rhodopsin (rods)
DAPI (nuclei)
a
host
transplant
60 dps
50 µm
b
host
host PR
transplant
50 µm
c
host PR
host
transplant
50 µm
RG opsin (cones)
SC-121 (donor)
Recoverin
(photoreceptors, cone bipolar cells)
Recoverin
DAPI (nuclei)
d
host
transplant
152 dps
50 µm
e
host
transplant
50 µm
f
host
transplant
50 µm

Supplemental Figure S1 (refers to Figures 5 and 6): Photoreceptor markers in transplants 2-5 months post-surgery

(a-c) Transplant 60d post-surgery (same transplant as in Fig. 4a,b). Note that there is still a thin host photoreceptor layer present. **(d-f)** Transplant 152d post-surgery (same transplant as in Fig. 4 c,d). Host photoreceptors have mostly disappeared. **(a,d)** Combination of SC-121 (red) with RG-opsin (green). **(b,e)** Recoverin (green). **(c,f)** Rhodopsin (red), DAPI (blue, nuclei). Scale bars = 50μm.
