# Supplementary material for: Retina Organoid Transplants Develop Photoreceptors and Improve Visual Function in RCS Rats With RPE Dysfunction
Source: Invest Ophthalmol Vis Sci. 2020 Sep 18;61(11):34. doi: 10.1167/iovs.61.11.34 (PMC7509771; doi:10.1167/iovs.61.11.34)
Supplement: Supplement 2 [file iovs-61-11-34_s002.pdf]

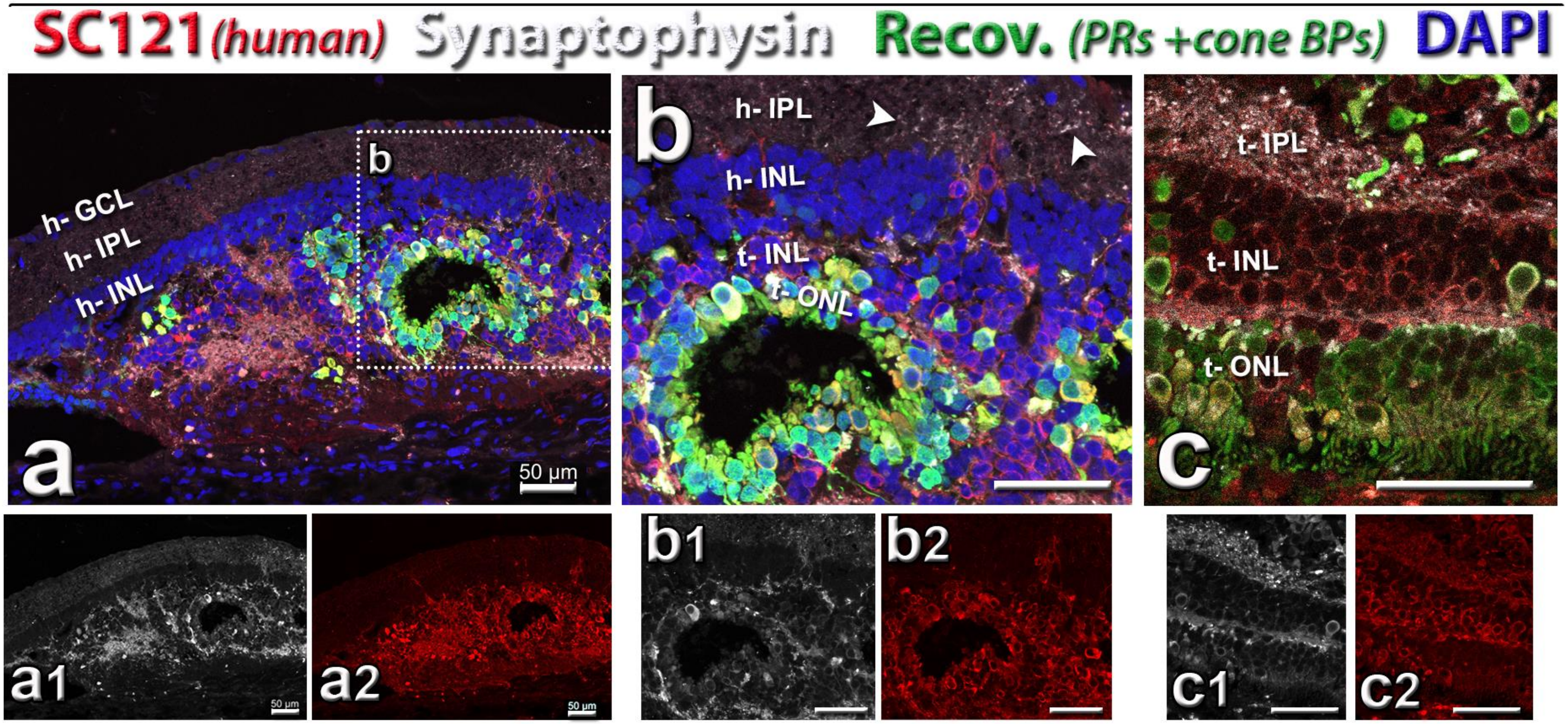

Supplemental Figure S2 (refers to Figure 7): **Triple staining for synaptophysin in combination with donor label SC121 and Recoverin**

(a-c): Combination of SC121 (human cytoplasm, red), synaptophysin (synapses, white), recoverin (photoreceptors and cone bipolar cells, green), and DAPI (nuclei, blue in a and b). **(a)** overview (showing subretinal transplant with large rosette (Transplant #4, same transplant as in Figure 4). **(b)** enlargement. Arrowheads point to strongly labeled synaptophysin puncta in host IPL close to transplant processes (SC121). Transplant bones in rosettes are strongly labeled for synaptophysin. **(c)** Enlarged area of Transplant #5 (same as in Figure 5) showing area with parallel layers (good lamination). Scale bars = 50 µm
