# Supplementary material for: Retina Organoid Transplants Develop Photoreceptors and Improve Visual Function in RCS Rats With RPE Dysfunction
Source: Invest Ophthalmol Vis Sci. 2020 Sep 18;61(11):34. doi: 10.1167/iovs.61.11.34 (PMC7509771; doi:10.1167/iovs.61.11.34)
Supplement: Supplement 3 [file iovs-61-11-34_s003.pdf]

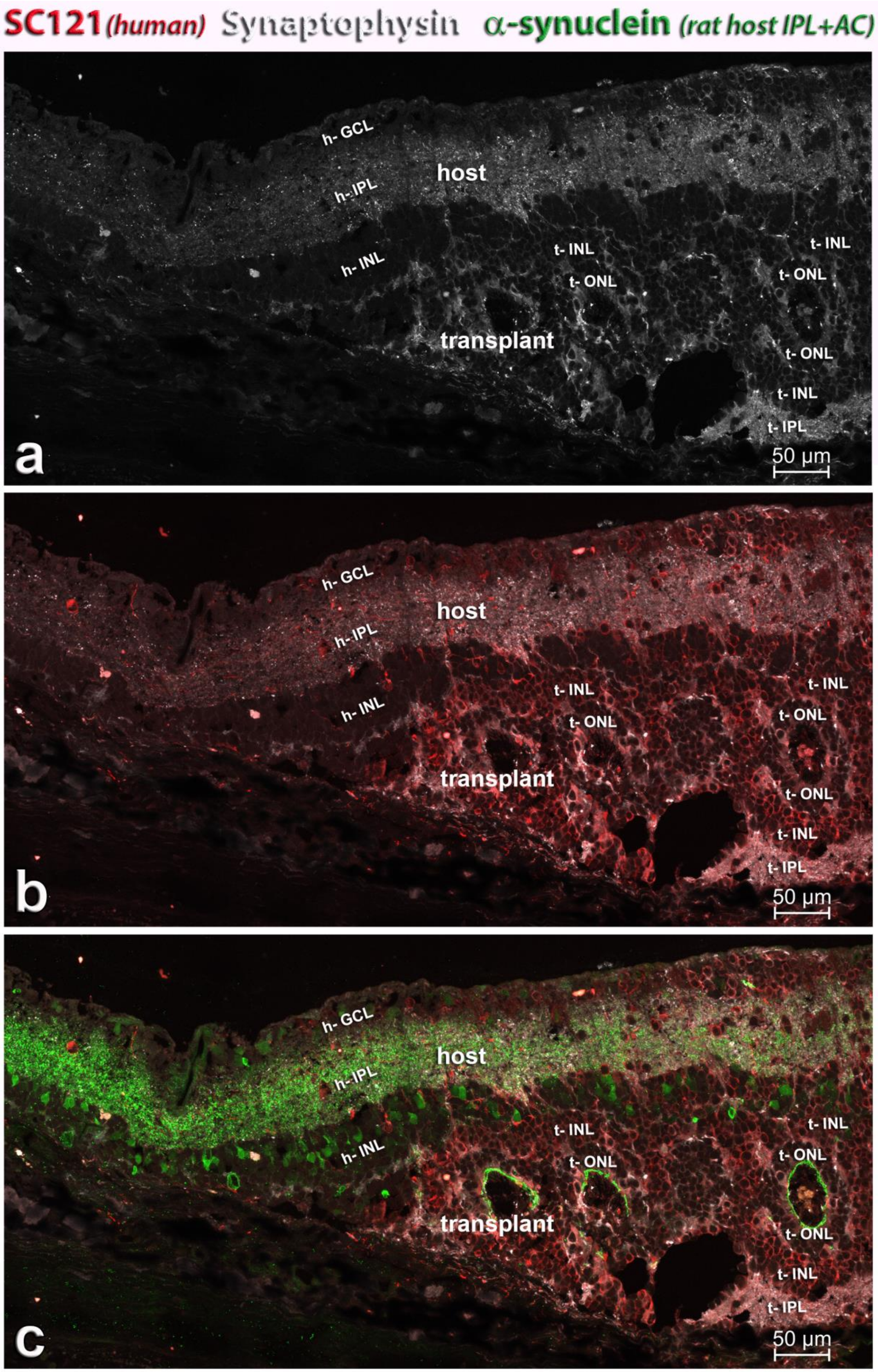
SC121 (human) Synaptophysin α-synuclein (rat host IPL+AC)
h- GCL
host
h- IPL
h- INL
t- INL
t- ONL
t- INL
t- ONL
transplant
t- ONL
t- INL
t- IPL
50 µm
a
b
c

Supplemental Figure S3 (refers to Figure 7): Synaptophysin in host over transplant versus outside

Combination of synaptophysin (white), the human cytoplasmic marker SC-121 (red), and rodent-specific α-synuclein (green). Confocal images of transplant #12 (see Table 3). **(a)** Synaptophysin alone. **(b)** Synaptophysin and SC-121. **(c)** Synaptophysin, SC-121 and rodent-specific α-synuclein. Note that synaptophysin immunoreactivity in the host IPL is much stronger over the transplant than outside. Scale bars = 50μm.
