# Supplementary material for: Retina Organoid Transplants Develop Photoreceptors and Improve Visual Function in RCS Rats With RPE Dysfunction
Source: Invest Ophthalmol Vis Sci. 2020 Sep 18;61(11):34. doi: 10.1167/iovs.61.11.34 (PMC7509771; doi:10.1167/iovs.61.11.34)
Supplement: Supplement 4 [file iovs-61-11-34_s004.pdf]

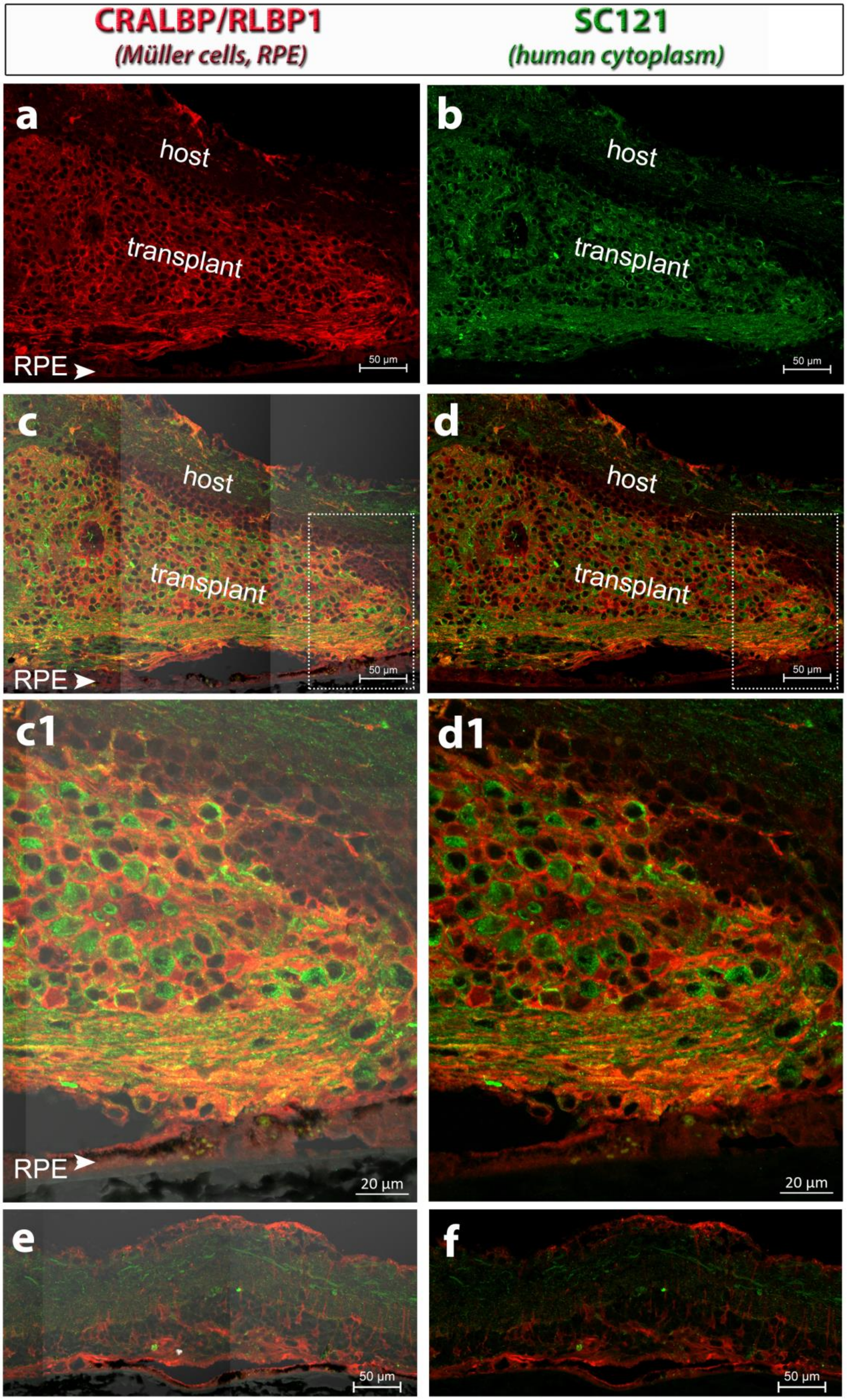

CRALBP/RLBP1
(Müller cells, RPE)
SC121
(human cytoplasm)
a
host
transplant
RPE
50 µm
b
host
transplant
50 µm
c
host
transplant
RPE
50 µm
d
host
transplant
50 µm
c1
RPE
20 µm
d1
20 µm
e
50 µm
f
50 µm

Supplemental Figure S4 (refers to Figure 9): Müller and RPE-specific marker CRALBP (RLBP1) in combination with human cytoplasm marker SC121. Confocal images of Transplant #5. **a)** RLBP1 (single channel,red). The transplant shows much stronger immunoreactivity for this RLBP1 antibody than the host retina. Host RPE at the bottom of the picture are only faintly stained. **b)** SC121 (single channel, green). **c)** Both channels and bright field. Box shows enlargement in c1. **d)** Both channels without bright field. Box shows enlargement in d1. **e,f)** host retina outside transplant shows SC-121 immunoreactive processes in inner plexiform layer. Host RPE show stronger RLBP1 immunoreactivity than under transplant.
