# Supplementary material for: Retina Organoid Transplants Develop Photoreceptors and Improve Visual Function in RCS Rats With RPE Dysfunction
Source: Invest Ophthalmol Vis Sci. 2020 Sep 18;61(11):34. doi: 10.1167/iovs.61.11.34 (PMC7509771; doi:10.1167/iovs.61.11.34)
Supplement: Supplement 5 [file iovs-61-11-34_s005.pdf]

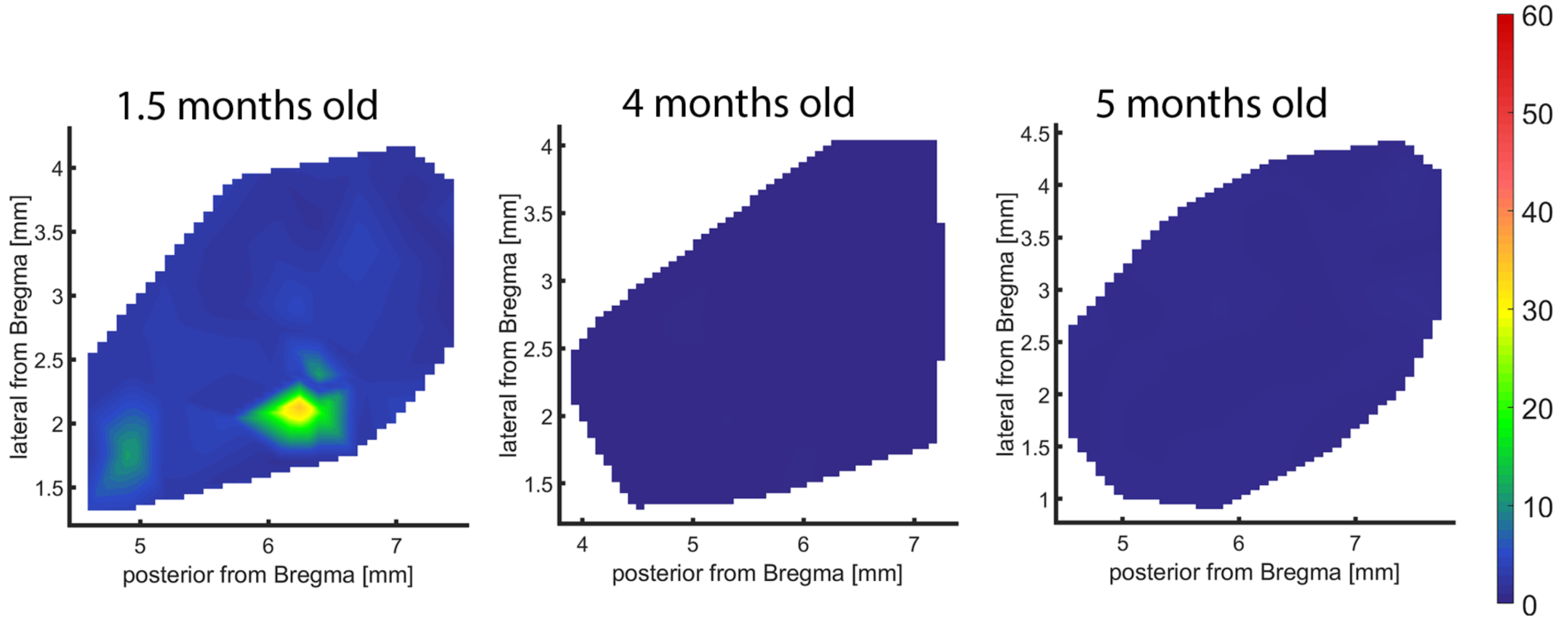

Supplemental Figure S5 (refers to Figure 12): SC recording data of non-surgery RCS rats between 1.5 and 5 mo. of age: Representative heatmaps of spike counts from 1.5 – 5 months old RCS rats. No visual responses could be recorded at the age of 3 months and later.
